# Evaluation of the Effects of Pasireotide LAR Administration on Lymphocele Prevention after Axillary Node Dissection for Breast Cancer: Results of a Randomized Non-Comparative Phase 2 Study

**DOI:** 10.1371/journal.pone.0156096

**Published:** 2016-06-09

**Authors:** Elisabeth Chéreau, Catherine Uzan, Emmanuelle Boutmy-Deslandes, Sarah Zohar, Corinne Bézu, Chafika Mazouni, Jean-Rémi Garbay, Emile Daraï, Roman Rouzier

**Affiliations:** 1 Department of Gynecology, Hôpital Tenon, Paris, France; 2 ED 394—Ecole Doctorale Physiologie Physiopathologie, Paris, France; 3 EA 3499 "Transporteurs ABC et épithéliums normaux et tumoraux," Paris, France; 4 Department of Surgery, Gustave Roussy, Paris, France; 5 Department of Biostatistics, Hôpital Saint Louis, Paris, France; 6 INSERM, U1138, team 22, University Paris 5, University Paris 6, Paris, France; 7 Department of Surgery, Institut Curie, Saint-Cloud, France; 8 EA 7285: Risques cliniques et sécurité en santé des femmes et en santé périnatale, Versailles-St-Quentin-en-Yvelines University, Montigny-le-Bretonneux, France; ACTREC (Advanced Centre for Treatment, Research and Education in Cancer) / Tata Memorial Centre, INDIA

## Abstract

**Objective:**

The aim of this study was to assess the efficacy (response rate centered on 80%) of a somatostatin analog with high affinity for 4 somatostatin receptors in reducing the postoperative incidence of symptomatic lymphocele formation following total mastectomy with axillary lymph node dissection.

**Setting:**

This prospective, double-blind, randomised, placebo-controlled, phase 2 trial was conducted in two secondary care centres.

**Participants:**

All female patients for whom mastectomy and axillary lymph node dissection were indicated were eligible for the study, including patients who had received neo-adjuvant chemotherapy. Main exclusion criteria were related to diabetes, cardiac insufficiency, disorder of cardiac conduction or hepatic failure.

**Interventions:**

Patients were randomised to receive one injection of either prolonged-release pasireotide 60 mg or placebo (physiological serum), which were administered intramuscularly 7 to 10 days before the scheduled surgery. The study was conducted in a double-blind manner.

**Primary and Secondary Outcome Measures:**

The primary outcome measure was the percentage of patients who did not develop post-operative axillary symptomatic lymphoceles during the 2 postoperative months. Secondary endpoints were the total quantity of lymph drained, duration and daily volume of drainage and aspirated volumes of lymph.

**Results:**

Ninety-one patients were randomised. Ninety patients were evaluable: 42 patients received pasireotide, and 48 patients received placebo. The mean estimated response rate were 62.4% (95% Credibility Interval [CrI]: 48.6%-75.3%) in the treatment group and 50.2% (95% CrI: 37.6%-62.8%) in the placebo group. Overall safety was comparable across groups, and one serious adverse event occurred. In the treatment group, one patient with known insulin-depe*ndent diabetes required hospitalization for hyperglycaemia.

**Conclusions:**

With this phase 2 preliminary study, even if our results indicate a trend towards a reduction in symptomatic lymphocele, pre-operative injection of pasireotide failed to achieve a response rate centered on 80%. Pharmacokinetics analysis suggests that effect of pasireotide could be optimised.

**Trial Registration:**

ClinicalTrials.gov NCT01356862

## Introduction

The principal morbidity following axillary lymph node dissection (ALND) for breast cancer is the postoperative development of lymphoceles following the removal of an axillary drain. This condition may result in pain, repeated lymphocele aspiration and infections and could delay local healing and adjuvant treatments. According to the literature, the incidence of lymphocele development can vary from 4 to 89% depending on the type of surgery, whether a drain is inserted or a compression dressing is applied and the time at which the drain is removed [1]. Many interventions have been used to decrease lymphocele formation after ALND, including the following: suction drains, compressive dressings, shoulder immobilization, fibrin glue, careful hemostasis and lymphostasis and axillary padding [2–8]. Despite all of these measures, lymphocele formation remains a frequent postoperative complication after ALND.

Octreotide, a somatostatin analog, has been successfully used for the medical management of postoperative gastrointestinal and pancreatic fistulae [9]. Two recent studies have demonstrated its value in reducing lymphoceles following ALNDs performed as part of breast cancer surgery [10, 11]. However, one recent study, published during our study, did not identify any significant reduction of lymphorrhea using lanreotide, another somatostatin analogue [12].

Pasireotide, a somatostatin analog registered for Cushing disease [13], that has a high affinity for somatostatin receptors (sst) (30 to 40 times greater for sst1 and sst5 receptors than octreotide, 5 times greater for sst3 and a slightly lower affinity than octreotide for sst2) is a promising molecule for this indication [14].

Based on the encouraging results published with octreotide and the greater effects anticipated with pasireotide, we sought to assess the benefits of a one time preoperative administration of pasireotide in reducing the incidence of axillary lymphocele formation following mastectomy-axillary node dissection.

The purpose of this trial was to assess whether pre-operative injection of pasireotide long-acting release (LAR) could reduce the incidence of symptomatic lymphocele formation following ALND.

## Methods

This prospective, double-blind, randomized, placebo-controlled, phase 2 trial was conducted in two French centers (Hopital Tenon, Paris and Gustave Roussy, Villejuif) from September 2010 to June 2012 (S1–S3 Files). This study was approved by the national ethics committee and French health authorities in April 2010 (CPP Ile de France IV) and registered in the EU Clinical Trials Register in May 2010 (N° EudraCT: 2010-018795-24) and in the ClinicalTrials.gov registry in 2011 (NCT01356862). Because of a change of the principal investigator there was a delay in registering this study (after enrolment of participants started) in ClinicalTrials.gov registry. The authors confirm that all ongoing and related trials for this intervention are registered. Written informed consent was obtained preoperatively in accordance with the ethical standards of the Helsinki declaration. The funders had no role in study design, data collection and analysis, decision to publish, or preparation of the manuscript. Protocol is available upon request to the corresponding author.

### Patients and treatment

All female patients for whom mastectomy and ALND were indicated were eligible for the study, including patients who had received neo-adjuvant chemotherapy. Main exclusion criteria were related to diabetes, cardiac insufficiency, disorder of cardiac conduction or hepatic failure. Others exclusion criteria are listed in Appendix 1. Patients were randomized to receive one injection of either prolonged-release pasireotide 60 mg or placebo (physiological serum), which were administered intramuscularly 7 to 10 days before the scheduled surgery. Randomizations were stratified on whether a neo-adjuvant treatment had been administered prior to surgery, overweight/obesity with a BMI>25 and the study center. This choice of these variables was decided because they could influence the rate of post-operative lymphocele and so created bias. Randomization was carried out by the Statistics Team at Hôpital Saint Louis in charge of the project (only one randomization list for both centers). The doctor who has included the patient contacted the randomization center which put in touch (telecopy) with the nurse who practiced the injection.

The study was conducted in a double-blind manner, i.e., neither the patients nor the treating surgeon were informed of the type of treatment administered.

Only the consultation nurse was informed of the treatment administered because pasireotide had to be suspended bedside just before injection. This nurse who was in charge of the suspension and the injection was never in contact with the surgeons because she was from another department and was never present during the patient's hospital stay or subsequent visits warranting aspiration, and the consultations were not held in the same location in either of the two study centers.

### Surgery and post-operative course

All patients underwent mastectomy with ALND. In each center, three surgeons were involved in this study. Hemostasis and lymphostasis were performed with clips and electrocautery. At the end of the surgery, two closed suction drains were left in the operative sites, always at the same place (the mastectomy site and the ALND site). Daily drainage volume was recorded during hospital stay or by a nurse at home if the patient had leave hospital with drains. The drains were removed when the daily drainage volume was less than 50 cc, and the maximum total drainage duration was 7 days. Both drains could be removed at the same time. The drain was with suction for the first four days and without suction starting on the 5th postoperative day.

### Endpoints

The primary endpoint of this study was the occurrence of post-operative symptomatic lymphocele, which was defined as the absence of aspiration or a unique or iterative aspiration global volume of lymphocele ≤ 60 cc in the 28 days after the intervention or a systematic aspiration volume ≤ 120 cc on the 28^th^ day.

Secondary endpoints were the total quantity of lymph drained up to the time of drain removal, duration of drainage, daily drainage volume, aspirated volumes of lymph, local healing, infections, fever, length of hospital stay and length of time to onset of adjuvant chemotherapy.

### Follow-up

The screening was performed during the pre-surgical visit. At the baseline visit (V1) 7 to 10 days prior to surgery, patients signed the informed consent, and pasireotide or the placebo were injected. In addition, a blood sample for pharmacokinetic monitoring was taken. On the day of surgery or the day after surgery (V2), data concerning the operation (type of surgery, duration, intraoperative complications, type and number of drains) and recording of adverse events were performed. On the day of discharge (V3), the summary of the daily collection of postoperative data was completed (daily and total drainage volumes, dates on which drains were removed and overall duration of drainage, postoperative complications, whether aspiration of a symptomatic lymphocele was required, length of hospital stay and recording of adverse events).

An immediate postoperative visit was required 14 ± 2 days following the surgery (V4) to record the number of repeated lymphocele aspirations and the volume (patients requiring an aspiration were seen during the interval between two consultations at the hospital, and data were collected in the medical file) and adverse events. A second blood sample for pharmacokinetic monitoring was taken during this visit.

The postoperative visit was 28 ± 4 days following surgery (V5), and a routine lymphocele aspiration was warranted and considered positive if the aspirated volume exceeded 120 cc. Again, data on the number of repeated lymphocele aspirations and the volume were collected, and adverse events were recorded.

Follow-up visits at 2 months (V6) allowed the recording of late adverse events.

**Fig 1:**
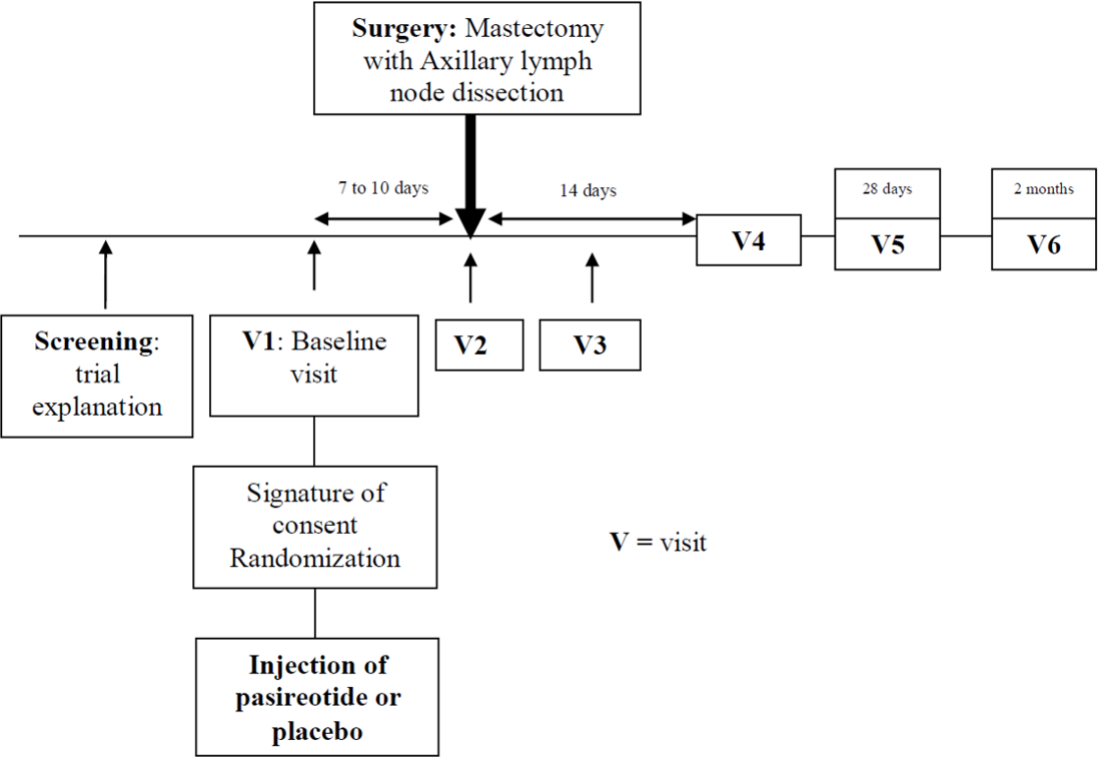
Trial design.

The design of this study is reported in Fig 1.

### Statistical methods

#### Bayesian sequential analysis

The aim of this phase 2, randomized, non-comparative trial was to assess the rate of patients who did not develop post-operative axillary symptomatic lymphoceles after receiving pasireotide or placebo (S4 File). The primary motivation for this sequential Bayesian strategy were: (1) the uncertainty about the effect of the procedure, (2) the small sample and (3) it allows continuous monitoring of outcomes throughout the trial. Moreover, as pointed out by Mandrakar and Sargent [15] single-arm phase II trial designs for evaluating an experimental procedure are limited by outcome-trial effect confounding arising from the inability to separate trial effects (selection biais, etc) from treatment effect on clinical outcomes. This is the reason why we decided to evaluate this procedure in a phase 2 randomized non comparative trial. The Bayesian paradigm provided a formal basis for learning about the response rate (defined as the patients who did not develop post-operative axillary symptomatic lymphoceles after receiving pasireotide or placebo) in each group as patient outcomes were observed and thus for making decisions on that basis [16, 17]. We used our experience with post-operative axillary symptomatic lymphoceles before the beginning of the trial, which was formalized as a prior distribution of the success rate centered on 80% in the treatment group and 60% in the placebo group. As the patient outcomes in the trial were recorded, the subsequent distribution of the outcome rate in each group was computed by applying Bayes’ theorem, which yielded a mean estimated success rate with a 95% credibility interval (CrI; measure of Bayesian precision, for additional details for the method part please see additional material). Additionally, based on the subsequent distribution experimental group, early stopping criteria were calculated to allow early termination of the trial for inefficacy if there was substantial evidence that the estimated response rate was unacceptably low compared to the expected minimal response rate of 60%. Results are expressed as medians and first and third quartiles [Q1-Q3] for quantitative data and counts and percentages for categorical data. Because of the design of the study (phase 2 with control group), the groups (placebo and treatment) were not compared.

Analyses were performed using R statistical package (online at http://www.R-project.org).

## Results

### Patients and surgical characteristics

**Fig 2:**
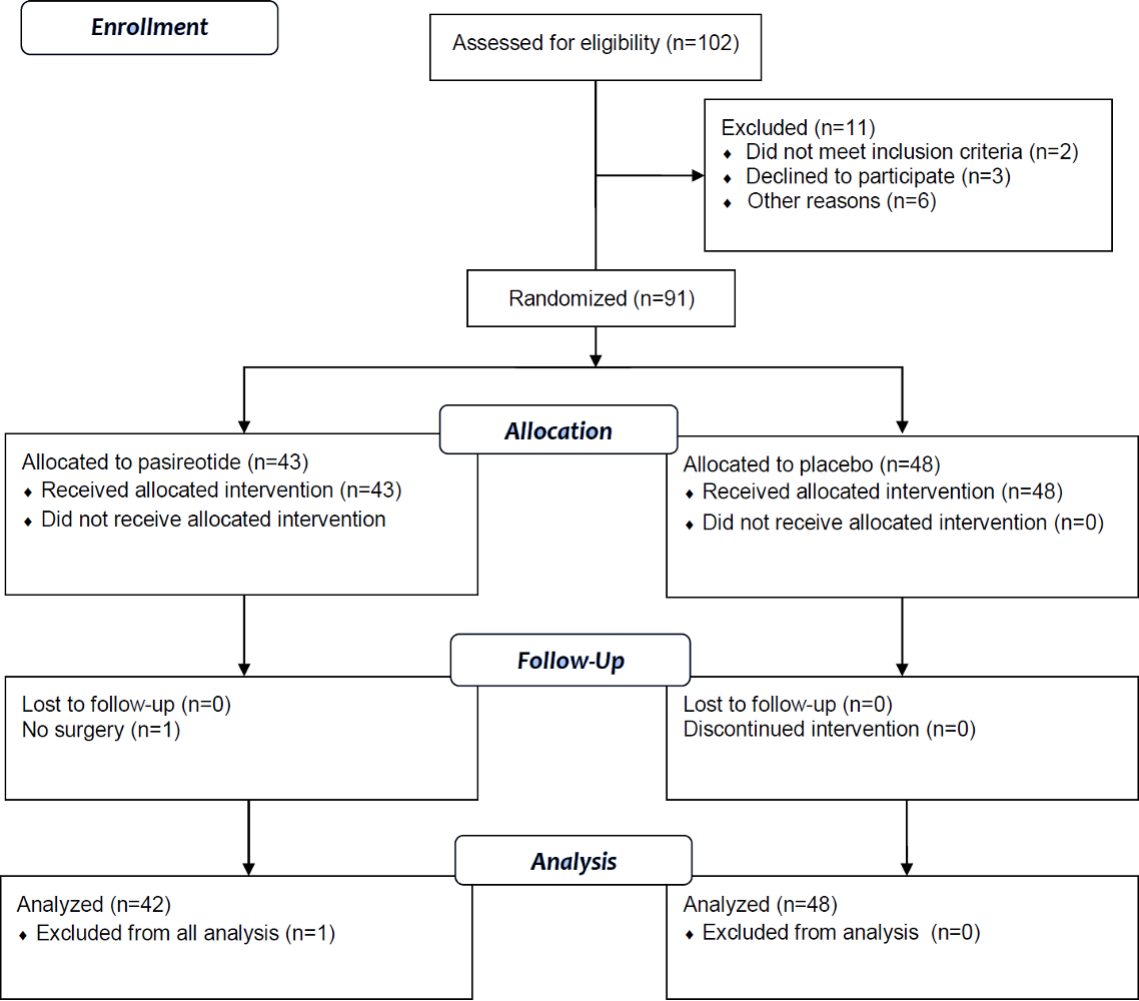
Flow chart of the trial.

Ninety-one patients were randomized over 18 months. The numbers of included patients were equal in the two centers (46 patients in Hopital Tenon and 44 patients in Gustave Roussy). Of these, 43 were randomized to the treatment group and 48 to the placebo group (Fig 2). One patient randomized to the pasireotide group was injected but not operated on because of an anaphylactic reaction to anesthesia, which delayed the surgery. The surgery was performed 3 weeks after the injection after anaphylactic tests were completed. Therefore, she was excluded from the analysis. For the analysis, 42 patients received pasireotide, and 48 patients received placebo (S5 File). At baseline, the patients’ characteristics were homogeneous in the two groups (Table 1).

**Table 1:** Patient and surgical characteristics.

N,%, Median, IQR	Pasireotide (n = 42)	Placebo (n = 48)
**Age (years)**	**54.5 [45,66.5]**	**51.5 [43,63]**
**BMI (kg/m**^**2**^**)**	**26.7 [23.9,29.3]**	**24.6 [21.8,29.4]**
**Obesity defined by a BMI > 25** *	**26 (62%)**	**20 (4%)**
**Diabetes (n,%)**	**3 (7%)**	**2 (4%)**
**Hypertension (n,%)**	**13 (31%)**	**10 (21%)**
**Post-menopausal (n,%)**	**27 (64%)**	**27 (56%)**
**Neo-adjuvant chemotherapy (n,%)** *	**15 (36%)**	**16 (33%)**
**Tumor size (mm)**	**25 [15,40]**	**25 [15,35]**
**Duration of surgery (min)**	**125 [91,155]**	**128 [93.75,155]**
**Tumor type**		
**Invasive ductal**	**35 (83%)**	**41 (85%)**
**Invasive lobular**	**6 (14%)**	**7 (15%)**
**Other**	**1 (2%)**	**0 (0%)**
**Compression bandage (n,%)**	**42 (100%)**	**47 (98%)**
**Number of patients with metastasis to lymph nodes (n,%)**	**24 (57%)**	**23 (48%)**
**Number of metastatic lymph nodes (n)**	**1 [0;2]**	**1 [0;2]**
**Number of lymph nodes removed (n)**	**11 [8,14.75]**	**15 [10,19]**
**Time between injection and surgery (days)**	**7 [7,8]**	**7 [7,8]**
**Inclusion center (n,%)** *		
**Hopital Tenon**	**21 (50%)**	**52%)**
I**nstitut Gustave Roussy**	**21 (50%)**	**23 (48%)**

BMI: Body Mass Index, IQR: Interquartile Range

*: variables used for a stratification

### Control group

**Fig 3:**
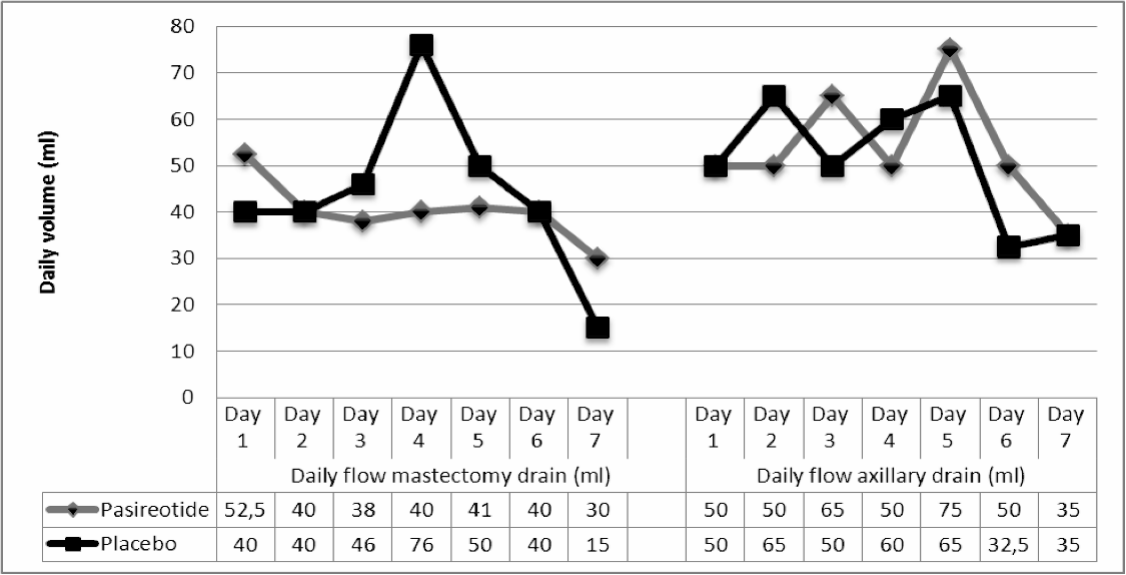
Daily drainage quantities.

The median tumor size was 25 mm, and the median surgical duration was 128 min. Fifteen patients had received neo-adjuvant chemotherapy prior to surgery, and 20 patients (42%) were considered obese (BMI>25), 62. All had compressive bandages at the end of the surgical course. The median time between treatment injection and surgery was 7 days. No patients were lost during the follow-up period. The estimated mean posterior success rate was 50.2% in the placebo group (95% CrI: 37.6%-62.8%) (Table 2, for additional computational details for Bayesian analysis please see additional material). The median duration of mastectomy and axillary drainages were 2 days and 3 days, respectively (Table 3 and Fig 3). Twenty-one patients required repeated lymphocele aspirations, with mean numbers of aspirations of 1.2. Fifteen patients underwent systematic lymphocele aspiration on day 28 (median volume: 210 ml). There were 5 wound-healing complications in the placebo group (4 cutaneous necroses and 1 abscess). The median delays between surgery and adjuvant chemotherapy was 49 days.

**Table 2:** Primary outcome results.

	Pasireotide arm	Placebo arm
**Prior distribution**	**Beta (5.6,1.4)**	**Beta (6.6,4.4)**
**N**	**42**	**48**
**Number of observed successes**	**25**	**23**
**Estimated mean posterior of success rate**	**62.4%**	**50.2%**
**95% Credibility Interval (CrI)**	**48.6%-75.3%**	**37.6%-62.8%**

**Table 3:** Post-operative courses.

	Pasireotide (n = 42)	Placebo (n = 48)
	Median[IQR] or n,%	
**Duration of drainage (days)**		
**Mastectomy drain**	**2.5 [1.75,4]**	**2 [1,4]**
**Axillary drain**	**3 [2,5]**	**3 [2,4]**
**Total flow (ml)**		
**Mastectomy drain**	**132.5 [55.5,226]**	**93 [30,221]**
**Axillary drain**	**165 [81,400.2]**	**162.5 [60,339.2]**
**Repeated lymphocele aspirations**		
**Number of patients (n, %)**	**17 (40%)**	**21 (44%)**
**Total volume (ml)**	**270 [135,415]**	**200 [162.5,260]**
**Incidence of postoperative febrile episodes (n, %)**	**1 (2%)**	**0 (0%)**
**Incidence of wound healing complications**		
**Hematoma requiring iterative surgery (n, %)**	**0 (0%)**	**0 (0%)**
**Cutaneous necrosis (n, %)**	**2 (5%)**	**4 (8%)**
**Cutaneous abscess (n, %)**	**1 (2%)**	**1 (2%)**
**Length of the hospital stay (days)**	**5 [4,6]**	**6 [4,6]**
**Length of time to onset of adjuvant chemotherapy (days)**	**54 [37.5,60]**	**49 [40,58.5]**

### Pasireotide group

Fifteen patients had received neoadjuvant chemotherapy prior to surgery, and 26 patients (62%) were considered obese (BMI>25), 62. The median tumor size was 25 mm, and the median surgical duration was 125 min. Except for one patient, all had compressive bandages at the end of the surgical course. The median time between treatment injection and surgery was 7 days. No patients were lost during the follow-up period.

#### Primary endpoint

The estimated mean posterior success rate (i.e., patients who did not experience a symptomatic lymphocele) was 62.4% in the Pasireotide group (95% CrI: 48.6%-75.3%) (Table 2, for additional computational details for Bayesian analysis please see additional material). The cessation criterion associated with inefficacy was not met; the probability that the response rate was lower than 60% in the treatment group after 42 inclusions was estimated at 0.354.

#### Secondary endpoints (Table 3 and Fig 3)

The median duration of mastectomy and axillary drainages were 2.5 days and 3 days, respectively. Seventeen patients required repeated lymphocele aspirations with mean numbers of aspirations of 1.4. Twelve patients in the treatment group underwent systematic lymphocele aspiration on day 28, with a median volume of 200 ml. The median delay between surgery and adjuvant chemotherapy was 54 days. There were 3 wound-healing complications.

#### Safety

There were no complications related to the injection of the treatment, and all patients had normal skin appearance at the site of the injection.

One patient had a chest skin infection related to an infected lymphocele, which required surgical excision. One serious adverse event occurred in a patient with pre-existing insulin-dependent diabetes, who required hospitalization for hyperglycemia. Pre-inclusion glycated hemoglobin was acceptable (8%). This patient also experienced a recurrent lymphocele, which necessitated repeated aspiration. During the hospitalization for hyperglycemia, the patient developed an infected seroma that required surgery.

Regarding non-serious adverse events, 3 patients in the pasireotide group had diarrhea, and 3 patients in the placebo group complained of constipation. All of the other non-serious adverse events could be related to the surgery (pain at the surgical site, impaired mobilization of arms, skin inflammation, bruising and other factors).

#### Pharmacokinetic results

**Fig 4:**
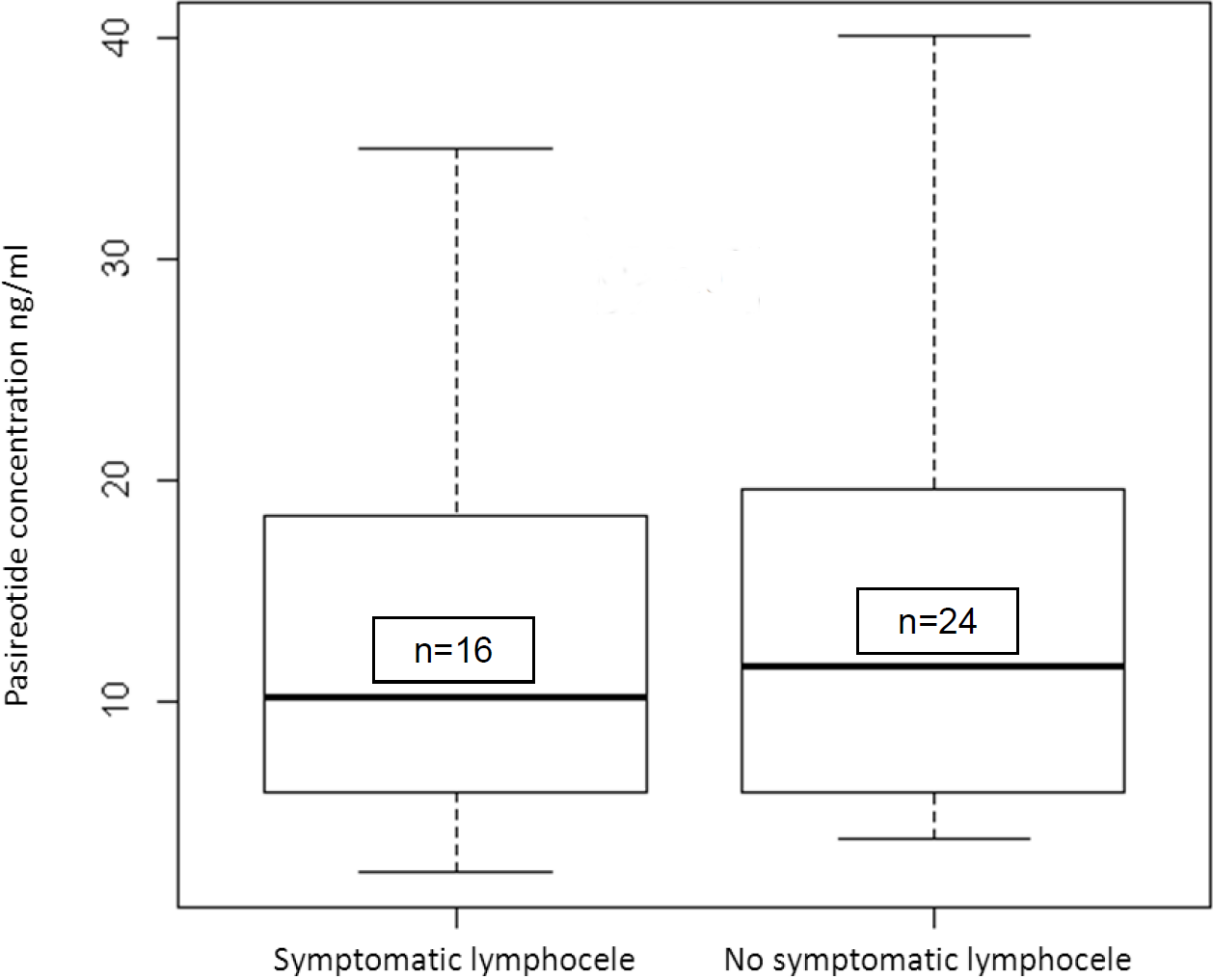
Comparison of mean pasireotide concentrations at day 14 in patients with or without post-operative symptomatic lymphocele formation.

We compared the pasireotide concentrations in blood samples taken 14 ± 2 days following surgery (V4) for pharmacokinetic monitoring between patients with and without symptomatic lymphoceles (Fig 4). No significant difference in the median pasireotide concentration was noted between the two groups (10.2 ng/ml vs. 11.65 ng/ml for failure or success, respectively).

## Discussion

Our phase 2, double-blind, randomized, placebo-controlled trial did not indicate a decrease in post-operative symptomatic lymphocele development with the pre-operative injection of pasireotide LAR.

Our data indicate a trend towards a reduction in symptomatic lymphocele formation with pasireotide. The estimated mean posterior response rate were 62.4% (95% CI: 48.6%-75.3%) in the treatment group and 50.2% (95% CI: 37.6%-62.8%) in the placebo group. Most of the risk factors for seroma formation, including body weight, age, number of lymph nodes removed, overweight and neo-adjuvant chemotherapy were controlled through randomization and stratification. The rate of symptomatic lymphocele formation in this trial was approximately 47%, which was similar to our preliminary study (40%). Although we failed to demonstrate a significant difference with our design, we detected a 12.2% decrease in symptomatic lymphocele development in treated patients. Based on these results, 550 patients would be necessary to demonstrate a significant difference in a phase 3 randomized study (275 patients who received placebo and 275 who received treatment, with alpha = 0.05 and beta = 0.2).

### Mode of action of pasireotide

Somatostatin is a widely distributed hormone in the nervous and gastropancreatic systems and is responsible for a variety of pharmacological and physiological effects. It can inhibit gastrointestinal endocrine and exocrine secretion and has anti-inflammatory action [18]. The direct effect of somatostatin on lymphatic flow has only been observed in the gastrointestinal tract.

Somatostatin receptors have been identified in lymphatic tissues, including those tissues not associated with the intestinal tract. Therefore, the inhibitory action of somatostatin on gastrointestinal lymphatic flow may also apply elsewhere in the body, particularly the lymphatic system [19]. Somatostatin may decrease lymphatic flow following axillary lymphadenectomy.

Several series have reported the use of octreotide in the treatment of chylous ascites or in the management of thoracic duct injury [20]. Although its mechanism of action has not been studied in depth, octreotide likely acts by inhibiting splanchnic blood flow and limiting the absorption of triglycerides.

Pasireotide is an injectable somatostatin analog. Similar to natural somatostatin and known analogs, its pharmaceutical efficacy depends on its binding to somatostatin receptors. The five somatostatin receptors (sst 1 to 5) are expressed in various organs under normal physiological conditions. Somatostatin analogs activate these receptors, which in turn reduce cell activity and inhibit hormone synthesis [21]. Octreotide and lanreotide, which are currently used, have strong affinities for the sst2 receptor and moderate affinities, if any, for the other receptor types. Pasireotide possesses greater affinity than octreotide for certain somatostatin receptors, specifically 30 times greater for sst1, 5 times greater for sst3 and 40 times greater for sst5. It possesses slightly lower affinity for sst2 and none (like octreotide) for sst4 [14].

The side effects described with pasireotide include potential transient, post-prandial, dose-dependent hyperglycemia, which mainly occurs after administration of a 600-μg dose. Intestinal adverse events have also been reported (diarrhea, nausea and vomiting) but do not generally warrant medication because they resolve spontaneously as treatment continues.

### Somatosatin analogues in lymphocele prevention

In addition to the many articles in the literature detailing the beneficial effects of octreotide on gastrointestinal and pancreatic fistulae and on regression of chylous ascites and chylothorax, a few articles have attempted to highlight the effect of octreotide on postoperative lymphoceles.

Two articles have highlighted the positive effects of somatostatin analogs on reducing drainage volumes and the incidence of lymphoceles following axillary node dissection in breast cancer. In 2003, Carcoforo *et al*. [10] reported a significant difference between a group of patients treated with subcutaneous octreotide during the immediate postoperative period and an untreated group. The treated group had smaller daily drainage volumes (65.4 vs. 94.6 ml, p = 0.0001), and their drains were removed earlier after surgery (7.1 vs. 16.7 days, p = 0.0001). Another more recent article by Mahmoud *et al*. [11] showed the same beneficial effects on the mean daily drainage volume (104 vs. 145 ml, p = 0.0001), the total duration of drainage (12.7 vs. 25 days, p = 0.0001) and the need for postoperative aspiration of lymphoceles (40 vs. 90%, p = 0.0001).

More recently, Gauthier *et al*. [12] have reported the results of a phase 3, double-blind, randomized study using Lanreotide Autogel 90 mg LAR to reduce lymphorrhea volumes in the axillary drain during the first 4 postoperative days. They showed a non-significant tendency towards a reduction of the lymphorrhea volume in the lanreotide group (median 292 ml, range 1–965 ml) compared to the placebo group (median 337 ml, range 0–1230 ml). In the group of patients who only underwent an axillary dissection, the lymphorrhea volume was significantly reduced in the lanreotide group (p = 0.035) compared to the placebo group. The absence of significant differences across the entire population could be explained by a lack of power and the fact that the authors included women with breast cancer who had to undergo ALND either alone or with either lumpectomy or mastectomy. To avoid the effects of breast surgery, we chose to include only patients who underwent mastectomy with ALND.

In 2006, in the renal transplantation field, an article by Capocasale *et al*. [22] demonstrated that lymphatic leakage following transplantation was of shorter duration in patients treated with octreotide. Furthermore, a lower incidence of lymphocele formation was observed after drain removal.

Differences between the results reported by Carcoforo [10] and Mahmoud [11] and our results could be partially explained. First, the clinical practices were different. In our practice, drains were removed before the seventh post-operative day, whereas in the studies by Carcoforo [10] and Mahmoud^11^ the mean duration of drainage was between 7 and 25 days. Second, the other authors both used octreotide 0.1 mg three times a day subcutaneously for 5 [10] or 7 [11] days, starting on the first postoperative day. In our study, we chose a long-acting release formulation of pasireotide (pasireotide LAR) with a single injection 7 to 10 days before surgery. This option was motivated by pharmacokinetic studies that showed that the effect was obtained from the seventh day after pasireotide LAR injection and remained for up to 30 days [21]. This study demonstrated that there were two peaks after injection of pasireotide LAR (40 and 60 mg). The first peak was observed on day 1 after administration, which was followed by a decrease in concentration from days 1 to 7 and a second slow-release peak with maximum concentrations at approximately day 20, after which pasireotide plasma concentrations gradually decreased. A pharmacokinetic analysis in neuroendocrine tumors demonstrated that the residual concentration at day 28 was 16.5 ng/mL, which is over the concentration observed at day 14 in our study. We cannot exclude that a better timing of injection would have optimize the effect of pasireotide LAR to prevent lymphocyst [23]. One single injection was easier for the patients than 15 to 21 subcutaneous injections. Finally, to limit bias due to the type of surgery performed, we included only mastectomies associated with ALND, unlike Gauthier *et al*. [12].

## Conclusion

Even if our results indicate a trend towards a reduction in symptomatic lymphocele, a one-time injection of pasireotide LAR is not efficient to prevent symptomatic lymphocele development in women undergoing mastectomy with axillary dissection. The optimal timing of pasireotide LAR administration remains controversial based on pharmacokinetics results. Further clinical studies are warranted.

## Appendix 1: Non-Inclusion Criteria

- Patient under the age of 18 years.
- Patient who does not understand French.
- Patient not covered by the French national health insurance system.
- Patient exhibiting one or more contraindications to anesthesia and surgery.
- Patient with a contra-indication to pasireotide.
- Refusal by the patient.
- Scheduled sentinel node procedure.
- Abnormal coagulation or curative anticoagulant treatment.
- Women of child-bearing potential without effective contraception.
- Pregnant or breast-feeding women.
- Poorly controlled diabetes (HbA1c > 8%).
- History of radiotherapy.
- Recurrent breast cancer.
- Patient with a congestive cardiac insufficiency (NYHA category III or IV), an instable angina pectoris, sustained ventricular tachycardia or ventricular fibrillation episodes or history of myocardial infarction during the last 6 months.
- Patient presenting an extension of QT interval (QT corrected according to the Fridericia formula (QTcF)) at the screening or baseline (predose) > 450msec.
- History of syncope or family history of sudden death or significant cardiac arrhythmia.
- Risk factors for torsades de pointes: hypokaliaemia, hypomagnesaemia, known structural or ischaemic cardiac disease, bradycardia (HR<55/min) or high grade AV block.
- Concomitant disease that could prolong QT or increase exposure to the study medication including dehydration, renal or hepatic impairment.
- Concomitant medication known to increase the QT interval.
- Patient with an hepatic pathology such as cirrhosis, chronic hepatitis active or persistent, or an elevation of ALAT rate, ASAT rate twice higher than the normal superior limit (NSL).
- Patient having leucocytes < 3x10^9^/L, Hb < 90% LIN, platelets < 100x10^9^/L.
- Patient having a pathology or medical history susceptible to interfere with the realization of the study or results evaluation according to the judgment of the investigator or the study monitor.
- Patient participating to another clinical trial with another molecule in study during the month before the first dose.
- Known oversensitivity to somatostatine analogs or another component of prolonged release pasireotide or prolonged release octreotide formulations.

## Supporting Information

S1 File Consort Checklist. (DOC)

S2 File Protocol in English. (DOC)

S3 File Protocol in French. (DOC)

S4 File Statistical method description. (DOCX)

S5 File Data. (XLSX)
