# Supplementary material for: Evaluation of the Effects of Pasireotide LAR Administration on Lymphocele Prevention after Axillary Node Dissection for Breast Cancer: Results of a Randomized Non-Comparative Phase 2 Study
Source: PLoS One. 2016 Jun 9;11(6):e0156096. doi: 10.1371/journal.pone.0156096 (PMC4900597; doi:10.1371/journal.pone.0156096)
Supplement: S2 File — (DOC) [file pone.0156096.s002.doc]

Evaluation of the effect of pasireotide LAR administration in the lymphocele prevention after axillary node dissection for breast cancer.

**PROSPECTIVE, RANDOMIZED PHASE 2 TRIAL**

**Sponsors:**

**Alliance pour la recherche en Cancérologie (APREC) (Alliance for Oncology Research), Hôpital Tenon (Tenon Hospital)**

**Coordinating investigators:**

**Professor Roman Rouzier, Dr. C. Mazouni, Dr. Catherine Uzan, Dr. Elisabeth Chéreau**

**Associate Investigators:**

**Professor S. Uzan, Dr. F. Rimareix, Professor E. Daraï, Dr. M. Ballester, Dr. S. Gouy, Dr. JR. Garbay**

**- Department of Obstetrics, Gynecology and Human Reproduction, Professor S. Uzan**

**Hôpital Tenon, AP-HP Paris hospitals**

**- Department of Surgery, Dr. D. Elias**

**Gustave Roussy Institute**

**Support: Novartis**

**Table of contents**

1. Introduction [8](#__RefHeading___Toc246224604)

1.1. Mode of action of pasireotide [8](#__RefHeading___Toc246224605)

1.2 Literature review [9](#__RefHeading___Toc246224606)

1.3 Somatostatin analogs and cancer [11](#__RefHeading___Toc246224607)

2- Study objectives [13](#__RefHeading___Toc246224608)

2.1 Primary objective [13](#__RefHeading___Toc246224609)

2.2 Secondary objectives [13](#__RefHeading___Toc246224610)

3. Study protocol [14](#__RefHeading___Toc246224611)

3.1. Global study design [14](#__RefHeading___Toc246224612)

3.1.1. Type of trial [14](#__RefHeading___Toc246224613)

3.1.2. Study design [14](#__RefHeading___Toc246224614)

3.2. Discussion of the type of study [17](#__RefHeading___Toc246224615)

3.3. Study population [17](#__RefHeading___Toc246224616)

3.3.1. Patient population [17](#__RefHeading___Toc246224617)

3.3.2. Inclusion and non-inclusion criteria [17](#__RefHeading___Toc246224618)

3.3.3. Interruption or discontinuation of treatment [19](#__RefHeading___Toc246224619)

3.4. Treatments [20](#__RefHeading___Toc246224620)

3.4.1. Investigational therapy and reference therapy [20](#__RefHeading___Toc246224621)

3.4.2. Treatment assignment [20](#__RefHeading___Toc246224622)

3.4.3. Blind procedure [21](#__RefHeading___Toc246224623)

3.4.4. Concomitant treatments [21](#__RefHeading___Toc246224624)

3.4.5. Treatment compliance [21](#__RefHeading___Toc246224625)

3.4.6. Treatment packaging and management [22](#__RefHeading___Toc246224626)

3.5. Visits and evaluations [22](#__RefHeading___Toc246224627)

3.5.1. Schedule [24](#__RefHeading___Toc246224628)

3.5.2. Visit schedule [24](#__RefHeading___Toc246224629)

3.5.3. Evaluation criteria [26](#__RefHeading___Toc246224630)

3.5.3.1. Endpoints [26](#__RefHeading___Toc246224631)

6. Statistical methods [28](#__RefHeading___Toc246224632)

6.1. Statistical methods to be used [28](#__RefHeading___Toc246224633)

6.2. Efficacy evaluation: [28](#__RefHeading___Toc246224634)

6.3. Safety assessment [28](#__RefHeading___Toc246224635)

6.4. Number of subjects [28](#__RefHeading___Toc246224636)

6.5. Primary endpoint analysis [28](#__RefHeading___Toc246224637)

6. 6. Rules for discontinuation [29](#__RefHeading___Toc246224638)

Summary of Protocol

1. **Background**

The principal morbidity following axillary node dissection within the scope of breast cancer surgery is the post-operative development of lymphocele. According to the literature, incidence can vary from 4 to 89% depending on the type of surgery, whether or not a drain is inserted or a compression dressing applied and the time at which the drain is removed… In our experience, the incidence is 40% [IGR (Gustave Roussy Institute) data focusing on 70 patients between November 2008 and February 2009].

Encouraging results in terms of reducing postoperative lymphoceles as well as drainage duration and volume using Octreotide have been recorded in two recent studies. A new molecule developed by Novartis Laboratories, namely pasireotide, is a somatostatin analog possessing strong affinity for several somatostatin receptors (30 to 40 times greater for sst1 and sst5, 5 times greater for sst3 and equivalent for sst2)

1. **Purpose**

The purpose of this trial is to assess the efficacy of a pre-surgical injection of pasireotide LAR in reducing the postoperative incidence of symptomatic lymphoceles following axillary node dissection.

The secondary objectives are to assess the efficacy of prolonged release pasireotide on the duration of postoperative drainage, the daily drainage volume, the total drainage volume, the number of repeated lymphocele aspirations and the volume, the total volume of lymph aspirated, the incidence of postoperative febrile episodes, the length of hospital stay, and the length of time to onset of adjuvant chemotherapy. It is also to assess the safety of prolonged release pasireotide.

1. **Trial design**

We recommend a prospective, randomized, two-center trial according to a Bayesian design with a control arm receiving a placebo.

1. **Trial population**

All female patients for whom mastectomy - axillary node dissection is indicated at the pre-surgical stage will be eligible for inclusion. Control population of an identical size: randomization 1:1.

1. **Study Procedure**

- Inclusion criteria: any female patient scheduled for breast surgery with mastectomy and axillary node dissection indicated at the pre-surgical stage.

- Exclusion criteria:

- Female patient under the age of 18 or

- Patient unable to understand French

- Patient must be affiliated to the French National Health Insurance System

- Refusal by the patient

- Scheduled sentinel node procedure

- Patient exhibiting one or more contraindications to anesthesia and surgery

- Pasireotide administration contra-indicated: abnormal coagulation

- Women able to conceive and not practising an effective method of birth control

- Pregnant or breast-feeding women

- Poorly controlled diabetes (HbA1c > 8%)

- History of radiotherapy

- Recurrent breast cancer

- Patient with a congestive cardiac insufficiency (NYHA category III or IV), an instable angina pectoris, sustained ventricular tachycardia or ventricular fibrillation episodes or history of myocardial infarction during the last 6 months.

- Patient presenting an extension of QT interval (QT corrected according to the Fridericia formula (QTcF)) at the screening or baseline (predose) > 450msec

- History of syncope or family history of sudden death or significant cardiac arrhythmia

- Risk factors for torsades de pointes: hypokaliaemia, hypomagnesaemia, known structural or ischaemic cardiac disease, bradycardia (HR<55/min) or high grade AV block

- Concomitant disease that could prolong QT or increase exposure to the study medication including dehydration, renal or hepatic impairment

- Concomitant medication known to increase the QT interval

- Patient with an hepatic pathology such as cirrhosis, chronic hepatitis active or persistent, or an elevation of ALAT rate, ASAT rate twice higher than the normal superior limit (NSL)

- Patient having leucocytes < 3x10^9^/L, Hb < 90% LIN, platelets < 100x10^9^/L

- Patient having a pathology or medical history susceptible to interfere with the realization of the study or results evaluation according to the judgment of the investigator or the study monitor

- Patient participating to another clinical trial with another molecule in study during the month before the first dose

- Known oversensitivity to somatostatine analogs or another component of prolonged release pasireotide or prolonged release octreotide formulations.

- **Inclusion and signature of the informed consent form prior to surgery.**

- 1 intramuscular injection of prolonged-release pasiretotide or placebo 7 to 10 days before surgery

- Collection of surgical and post-surgical data (onset of a lymphocele, need for aspiration, number and volume of each repeated aspiration, daily drainage volume, duration of drainage, local infectious complications or delayed healing).

1. **Primary and secondary endpoints**

-The primary endpoint of this study is the patient ratio who did not have post-operative axillary symptomatic lymphoceles defined as the absence of aspiration or a unique or iterative aspirations global volume of lymphoceles inferior to 60cc inclusive (≤) in the 28 days after the intervention or a systematic aspiration volume at the 28^th^ day inferior to 120cc inclusive (≤).

- Secondary endpoints: total quantity of lymph drained on D5 and up to removal of drains, duration of drainage, daily drainage volume, aspirated volumes of lymph in the case of aspiration, local healing, infections, fever, length of hospital stay, length of time to onset of adjuvant chemotherapy.

1. **Statistical analysis and trial design**

The statistical analysis will be carried out sequentially after observing the principal criterion (i.e. success is defined as a total volume of lymphocele following single or repeated aspiration ≤ 60 cc in the 28 days following surgery or a routine aspiration volume on the 28^th^ day ≤ 120cc) of each patient included for each randomization group, with or without treatment.

It involves estimating the probability of a response in each group (proportion of patients who will not have undergone lymphocele aspiration or the aspiration of a total volume of ≤ 60cc or ≤ 120cc for the aspiration carried out on the 28^th^ day after surgery) using a Bayesian design based on a beta-binomial model. With the Bayesian approach, the response rate in each group (π*_i_*) is considered as a random variable, with *a priori* density focused on the anticipated response rate of 80% in the group receiving treatment and 60% in the non-treatment group, which will be sequentially updated as the observations are made according to a so-called *a posteriori* law.

A total of 90 patients will be included over 12 months with 45 patients in the treatment group and 45 patients in the non-treatment group.

# 1. Introduction

Octreotide, a somatostatin analog, has demonstrated its efficacy in the medical management of postoperative gastro-intestinal and pancreatic fistulae. Two recent studies have shown its value in reducing lymphoceles following axillary node dissection performed as part of breast cancer surgery.

The principal morbidity following axillary node dissection within the scope of breast cancer surgery is the postoperative development of lymphocele following the removal of an axillary drain. This may be a source of pain, repeated aspiration, infection and delayed local healing.

Pasireotide, a somatostatin analog under evaluation and possessing a high affinity for somatostatine receptors (30 to 40 times greater for sst1 and sst5 receptors, 5 times greater for sst3 and equivalent for sst2) is an attractive molecule in this indication.

Based on the encouraging results published with octreotide and the greater effects anticipated with pasireotide, we want to assess the benefit of preoperative administration of pasireotide in reducing the incidence of axillary lymphoceles following mastectomy - axillary node dissection.

## 1.1. Mode of action of pasireotide

Somatostatin is a hormone that is widely distributed in the nervous and gastropancreatic system responsible for a variety of pharmacological and physiological effects. It can inhibit gastrointestinal endocrine and exocrine secretion and has an anti-inflammatory action (1). The direct effect of somatostatin on lymphatic flow has only been observed on the gastrointestinal tract.

Several series report the use of octreotide in the treatment of chylous ascites or in the management of thoracic duct injury (2). Although its mechanism of action has not been studied in depth, it probably acts by inhibiting splanchnic blood flow and limiting the absorption of triglycerides.

Somatostatin receptors have been demonstrated in lymphatic tissues, including those not associated with the intestinal tract. It is therefore probable that the inhibitory action of somatostatin on gastrointestinal lymphatic flow may also apply elsewhere in the body, and to the lymphatic system in particular.

Somatostatin might therefore decrease lymphatic flow following essentially axillary lymphadenectomy.

A new molecule, pasireotide, is currently in clinical development. This molecule is a somatostatin analog possessing an affinity 30 to 40 times greater for sst1 and sst5 receptors, 5 times greater for sst3 and comparable for sst2 than octreotide. It could therefore have a greater effect than octreotide.

The gradual release form must be injected intramuscularly prior to surgery. An effective plateau concentration is obtained after 10 days regardless of the selected dosage (20, 40, or 60 mg) (3).

## 1.2 Literature review

In addition to the many articles in the literature detailing the beneficial effects of octreotide on gastrointestinal and pancreatic fistulae and on regression of chylous ascites and chylothorax, a few articles have attempted to highlight its effect on postoperative lymphoceles.

Firstly, two articles have highlighted the positive effect of somatostatin analogs in reducing drainage volume and the incidence of lymphoceles following axillary node dissection in breast cancer:

- In 2003 Carcoforo et al. (4) revealed a significant difference between a group of patients treated with subcutaneous octreotide during the immediate postoperative period and an untreated group. The treated group had smaller drainage volumes (65.4 vs. 94.6 ml, p=0.0001) and their drains were removed sooner after surgery (7.1 vs. 16.7 days, p=0.0001).
- Another more recent article by Mahmoud et al. (5) showed the same beneficial effects on the mean daily drainage volume (104 vs 145 ml, p=0.0001), the total duration of drainage (12.7 vs 25 days, p=0.0001) and the need for postoperative aspiration of lymphoceles (90 vs 40%, p=0.0001).

In 2006, in a different field (that of renal transplantation), an article by Capocasale et al. (6) showed that lymphatic leakage following transplantation was of shorter duration in the octreotide-treated group. Furthermore, a lower incidence of lymphocele was observed after drain removal.

Pasireotide is an injectable somatostatin analog. Like natural somatostatin and known analogs, its pharmaceutical efficacy depends on its binding to somatostatin receptors. There are five of these (sst 1 to 5); they are expressed in various bodily tissues under normal physiological conditions. Somatostatin analogs activate these receptors, which in turn reduce cell activity and inhibits hormone synthesis. (7). Octreotide and lanreotide, which are currently used, have a strong affinity for the sst2 receptor and moderate affinity, if any, for the other types. Pasireotide possesses greater affinity than octreotide for certain somatostatin receptors: 30 times greater for sst1, 5 times greater for sst3 and 40 times greater for sst5. It possesses equivalent affinity for sst2 and none (like octreotide) for sst4. (8)

The side effects described with pasireotide include potential transient, post-prandial, dose-dependent hyperglycemia, which mainly appears as from the administration of injections of 600μg. Intestinal disorders have been reported (diarrhoea, nausea and vomiting) but do not generally warrant medication since they spontaneously disappear as treatment continues.

In the literature, the incidence of axillary lymphoceles following surgery for breast cancer varies considerably: a recent meta-analysis of 66 studies revealed an incidence of 4 to 89 % depending on whether or not drainage was being carried out, the type of surgery (conservative or not), the time at which the drain was removed and whether or not a compression dressing was applied.

In our experience, the incidence at Hôpital Tenon is 40%. This percentage has been corroborated by Gustave Roussy Institute data, which recorded an incidence of 39.3% in out of 70 patients who underwent surgery between November 2008 and February 2009.

## 1.3 Somatostatin analogs and cancer

# Several teams have reported their use of Sandostatin® or other somatostatin analogs in the management of patients suffering from inoperable peritoneal carcinoma (9-12). This treatment was used as supportive therapy and reduced the incidence of symptoms associated with an obstructive syndrome. It fact, it can reduce gastrointestinal secretions. Some authors have even suggested that somatostatin analogs possess an anti-tumor effect (9). Octreotide is recommended by AFSSAPS in the treatment of palliative care patients presenting with intestinal occlusion in peritoneal carcinoma. Since a Marketing Authorization (MA) has been granted for the use of somatostatin analogs in patients presenting with a gastrointestinal endocrine tumor, the use of pasireotide does not raise any specific ethical issue (13).

# 2- Study objectives

## 2.1 Primary objective

The primary objective of this study is to assess the efficacy of a preoperative prolonged release pasireotide injection in the reduction in the incidence of symptomatic, postoperative axillary lymphoceles following mastectomy-axillary node dissection.

## 2.2 Secondary objectives

The secondary objectives of this study are:

- to evaluate the efficacy of prolonged release pasireotide on:

- the duration of postoperative drainage
- the daily drainage volume
- the total drainage volume
- the number of repeated lymphocele aspirations and the volume.
- the total volume of lymph aspirated
- the incidence of postoperative febrile episodes
- the length of the hospital stay
- the length of time to onset of adjuvant chemotherapy

- to evaluate the safety of prolonged release pasireotide

# 3. Study protocol

## 3.1. Global study design

### 3.1.1. Type of trial

Randomized, multicenter, prospective, Bayesian trial with a control group.

Since the control group receives a placebo injection, it can be used to validate the incidence of lymphocele following mastectomy and axillary node dissection during the study period (the incidence varies considerably according to the literature). It can be used to calculate the study number if a decision is taken to carry out a phase III study.

### 3.1.2. Study design

- The J_i_ data correspond to the length of time compared to the injection of pasireotide or placebo [J_1_ = day of injection], K_i_ data correspond to the duration of the procedure [K_0_=day on which surgery is performed].
- Screening: reporting and presurgical visit
  - Check for compliance with inclusion and non-inclusion criteria
  - Explanation of the trial to the patients
  - Issue of the Patient Information Letter
- Baseline visit (V1 = J_1_) 7 to10 days before surgery (K_-10_ to K_-7_):
  - Consent form signed
  - Injection of pasireotide or placebo by a consultation nurse who will not be present during subsequent visits since aspiration may be required.
  - Recording of adverse events
  - Blood sample for pharmacokinetic monitoring
  - ECG
- Visit V2 (day of surgery or day after surgery [K_0_ or K_1_]): collection of data concerning the operation (type of surgery, duration, intraoperative complications, type and number of drains)
  - Recording of adverse events:
- Visit V3 (day of discharge: K_4_ to K_7_ post-surgery): Summary of the daily collection of postoperative data:
  - The daily and total drainage volumes
  - Date on which drains were removed and overall duration of drainage
  - Postoperative complications (hematoma, skin necrosis, etc.)
  - Whether aspiration of a symptomatic lymphocele was required
  - Length of hospital stay
  - Recording of adverse events:
- Immediate postoperative visit (V4): 14+/- 2 days following surgery (K_12_ to K_16_)
  - The number of repeated lymphocele aspirations and the volume (patients requiring an aspiration will be seen during the interval between two consultations at the hospital and data will be colligated in the medical file. To ensure the lymphoceles sufficiency, a free phone number will be given to the patient in order that she could report any event if necessary.)
  - Recording of adverse events
  - ECG
  - Blood sample for pharmacokinetic monitoring
- Postoperative visit (V5): 28+/- 4 days following surgery (K_24_ to K_32_)
  - Routine lymphocele aspiration is considered positive if the volume aspirated exceeds 120cc.
  - The number of repeated lymphocele aspirations and the volume (patients requiring an aspiration will be seen during the interval between two consultations at the hospital and data will be colligated in the medical file.
  - Adjuvant chemotherapy start date, if required
  - Recording of adverse events
- Follow-up visits at two months (V6)
  - Recording of adverse events

**Figure 1: Trial design**

14 d

**Screening**: trial explanation

**V1**: Baseline visit

Signature of consent

Randomization

**Surgery:** Axillary node dissection

**V4**

**V5**

28 d

7 to 10 d

**V =** visit

**Injection of pasireotide or placebo**

**V2**

**V3**

**V6**

2 months

## 3.2. Discussion of the type of study

We want to assess the efficacy of prolonged release pasireotide because its safety has already been demonstrated and is well known (13). A phase 3 study is irrelevant in the absence of numerical efficacy data. The incidence of symptomatic lymphocele aspiration following mastectomy - axillary node dissection varies according to the literature and although our initial experience suggests an incidence rate of 40%, a control group is required. This is why we recommend a randomized phase II study.

## 3.3. Study population

### 3.3.1. Patient population

All female patients for whom mastectomy - axillary node dissection is indicated at the pre-surgical stage will be eligible for inclusion including patients who have received neo-adjuvant chemotherapy.

### 3.3.2. Inclusion and non-inclusion criteria

**Inclusion Criteria:**

- Female patient aged 18 years or over.
- Patient understands French.
- Patient covered by the French national health insurance system.
- Any female patient scheduled for breast surgery with mastectomy and axillary node dissection indicated at the pre-surgical stage.

**Non-inclusion Criteria:**

- Patient under the age of 18 years.
- Patient who does not understand French.
- Patient not covered by the French national health insurance system.
- Patient exhibiting one or more contraindications to anesthesia and surgery.
- Patient with a contra-indication to pasireotide
- Refusal by the patient
- Scheduled sentinel node procedure
- Abnormal coagulation or curative anticoagulant treatment
- Women of child-bearing potential without effective contraception,
- Pregnant or breast-feeding women
- Poorly controlled diabetes (HbA1c > 8%)
- History of radiotherapy
- Recurrent breast cancer
- Patient with a congestive cardiac insufficiency (NYHA category III or IV), an instable angina pectoris, sustained ventricular tachycardia or ventricular fibrillation episodes or history of myocardial infarction during the last 6 months.
- Patient presenting an extension of QT interval (QT corrected according to the Fridericia formula (QTcF)) at the screening or baseline (predose) > 450msec
- History of syncope or family history of sudden death or significant cardiac arrhythmia
- Risk factors for torsades de pointes: hypokaliaemia, hypomagnesaemia, known structural or ischaemic cardiac disease, bradycardia (HR<55/min) or high grade AV block
- Concomitant disease that could prolong QT or increase exposure to the study medication including dehydration, renal or hepatic impairment
- Concomitant medication known to increase the QT interval
- Patient with an hepatic pathology such as cirrhosis, chronic hepatitis active or persistent, or an elevation of ALAT rate, ASAT rate twice higher than the normal superior limit (NSL)
- Patient having leucocytes < 3x10^9^/L, Hb < 90% LIN, platelets < 100x10^9^/L
- Patient having a pathology or medical history susceptible to interfere with the realization of the study or results evaluation according to the judgment of the investigator or the study monitor
- Patient participating to another clinical trial with another molecule in study during the month before the first dose
- Known oversensitivity to somatostatine analogs or another component of prolonged release pasireotide or prolonged release octreotide formulations.

### 3.3.3. Interruption or discontinuation of treatment

The date and reason for withdrawal from the study or early treatment discontinuation must be entered in the Case Report Form. The investigators must make every effort to ensure that patients continue to participate and take the study medication throughout the trial. A final evaluation will be carried out and documented in the corresponding section of the Case Report Form (visit and study completion page) in the event of withdrawal from the study or early treatment discontinuation.

Patients may be withdrawn from the study for one of the following reasons:

- adverse, intolerable event(s),
- protocol violation, appearance of a protocol non-inclusion criterion,
- withdrawal of consent,
- patient lost to follow-up,
- administrative problem,
- death,

Any patient who withdraws from the study due to an adverse event or laboratory abnormality must be followed up until this adverse event has been resolved or the laboratory abnormality has returned to normal. Patients who are early withdrawn from the study will not be replaced.

## 3.4. Treatments

### 3.4.1. Investigational therapy and reference therapy

We want to test prolonged release pasireotide 60 mg.

No treatment for the prevention of postoperative axillary lymphoceles has been granted a marketing authorization to date.

The control group will receive an intramuscular placebo injection (physiological serum).

### 3.4.2. Treatment assignment

Patients will be randomized to receive either an injection of prolonged release pasireotide 60 mg or a placebo injection (physiological serum), which will be administered intramuscularly 7 to 10 days before scheduled surgery.

Randomization will be stratified on

1. whether or not a neo-adjuvant treatment has been administered prior to surgery.
2. overweight/obesity with a BMI > 25
3. the including center

### 3.4.3. Blind procedure

The procedure will be conducted double blind: neither the patients nor the surgeon in charge of the patients will be informed of the type of treatment administered.

Only the consultation nurse will be informed of the treatment administered because pasireotide is suspended prior to injection. On the other hand, this nurse will not be present during the patient's hospital stay or subsequent visits warranting aspiration. In fact, these consultations are not held in the same location in either of the two including centers.

Randomization will be carried out by the Statistics Team at Hôpital Saint Louis in charge of the project (only one randomization list for both centers). The doctor who has included the patient will contact the randomization center which will put in touch (telecopy) with the nurse who will practice the injection. Thus, this last one will reconstitute the pasireotide or prepare the placebo.

### 3.4.4. Concomitant treatments

Other treatments received by the patient during her hospital stay will be listed and attached to the CRF.

### 3.4.5. Treatment compliance

Since treatment comprises a single injection adminstered at the hospital by a state registered nurse, there are no treatment compliance issues.

### 3.4.6. Treatment packaging and management

Pasireotide will be provided by Novartis® Laboratory. The medicinal product will be repackaged and re-labelled for this study by an external service provider (LC2) and then redistributed to each center depending on requirements and randomization strategies.

## 3.5. Visits and evaluations

| ***Tests*** | ***Screening*** | ***Inclusion*** | **Day** | | | **Month** | |
| --- | --- | --- | --- | --- | --- | --- | --- |
| *Visits* |  | ***V1 = D1*** | ***V2*** | ***V3*** | **V4**^8^ | **V5** | **V6** |
| *Days* |  | *K_-10_ to K_-7_* | *K_0_ - K_1_* | *K_4_ to K_7_* | K_12_ to K_16_ | K_24_ to K_32_ | K_60_ |
| *Trial information* | X |  |  |  |  |  |  |
| *Inclusion - non-inclusion criteria* | X |  |  |  |  |  |  |
| *Informed consent form* |  | X |  |  |  |  |  |
| *Randomization* |  | X |  |  |  |  |  |
| *Demographic data, history of the tumor, medical and surgical history^1^*. |  | X |  |  |  |  |  |
| *Neodjuvant treatment* |  | X |  |  |  |  |  |
| *Injection of prolonged release pasireotide or placebo* |  | X |  |  |  |  |  |
| *Clinical evaluation, signs and symptoms ^2^* | X | X | X | X | X | X | X |
| *Adverse events ^3^* |  | X | *X* | *X* | *X* | *X* | *X* |
| *Surgical data ^4^* |  |  | X |  |  |  |  |
| *Postoperative data ^5^* |  |  |  | X |  |  |  |
| *Clinical evaluation ^6^* |  |  |  | X | X | X | X |
| *Permanent anatomopathological characteristics and adjuvant treatments ^7^* |  |  |  |  |  | X |  |
| *Routine lymphocele aspiration* |  |  |  |  |  | X |  |
| *Laboratory blood tests (pharmacokinetics)* |  | X |  |  | X |  |  |
| *ECG* |  | X |  |  | X |  |  |

1: demographic data (age, BMI), tumor history, medical and surgical history (breast surgery), concomitant medical diseases (diabetes, obesity), neo-adjuvant chemotherapy, tumor characteristics (TNM classification, histology, location)

2: weight, blood pressure, heart rate, fever.

*3: examination for skin reactions at the injection site (redness, pain, necrosis) and other adverse events.*

*4: type of procedure, duration, complications during surgery, compression dressing, type, number and location of drains.*

*5: daily and total drainage volume, date on which drains are removed and total drainage volume, postoperative complications (hematoma, skin necrosis, abscess, fever), presence of a lymphocele, lymphocele aspiration and duration of hospital stay.*

*6: number of lymphocyte aspirations, volume aspirated on each occasion, adjuvant chemotherapy start date. Skin test: redness, delayed healing, abscess, leakage.*

*7: histological type, grade, hormone receptors, Ki67, Her2, size of tumor, number of nodes removed, number of positive nodes, multifocal tumour, selected adjuvant treatments (chemotherapy and type of chemotherapy, number of cycles, hormone therapy, Herceptin® and radiotherapy)*

*8: If the V4 visit corresponds to more than 24 days after injection, this visit will be advanced in order to perform ECG and pharmacokinetic between 19 and 23 days after injection of pasiréotide LAR.*

### 3.5.1. Schedule

- Screening: reporting and presurgical visit
- Baseline visit (V1) 7 to 10 days before surgery
- Visit V2 (K_0_ or K_1_ postoperative)
- Visit V3 (day of discharge: K_4_ - K_7_ postoperative)
- Immediate postoperative visit (V4): 14+/- 2 days following surgery
- Postoperative visit (V5): 28+/- 4 days following surgery
- Follow-up visits at two months (V6)

### 3.5.2. Visit schedule

- Screening: reporting and presurgical visit
  - Check for compliance with inclusion and non-inclusion criteria
  - Explanation of the trial to the patients
  - Issue of the Patient Information Letter
- Baseline visit (V1) 7 to 10 days before surgery:
  - Consent form signed
  - Injection of pasireotide or placebo
  - Recording of adverse events
  - ECG
  - Blood sample for pharmacokinetic monitoring
- Visit V2 (K_0_ or K_1_ postoperative): collection of data concerning the operation (type of surgery, duration, intraoperative complications, type and number of drains)
  - Recording of adverse events:
- Visit V3 (day of discharge: K_4_ - K_7_ postoperative) Summary of the daily collection of postoperative data:
  - The daily and total drainage volumes
  - Date on which drains were removed and overall duration of drainage
  - Postoperative complications (hematoma, skin necrosis, etc.)
  - Whether aspiration of a symptomatic lymphocele was required
  - Length of hospital stay
  - Recording of adverse events:

It has been decided that drains will be removed when the daily drainage volume is less than 50 cc and that the maximum total duration of drainage will be 7 days. The drain will be emptied as from the 5^th^ postoperative day.

- Immediate postoperative visit (V4): 14+/- 2 days following surgery. If the V4 visit corresponds to more than 24 days after injection, this visit will be advanced in order to perform ECG and pharmacokinetic between 19 and 23 days after injection of pasiréotide LAR.
  - The number of repeated lymphocele aspirations and the volume (patients requiring an aspiration will be seen during the interval between two consultations at the hospital and data will be colligated in the medical file. To ensure the lymphoceles sufficiency, a free phone number will be given to the patient in order that she could report any event if necessary.)
  - Recording of adverse events
  - Blood sample for pharmacokinetic monitoring
  - ECG
- Postoperative visit (V5): 28+/- 4 days following surgery
  - Routine lymphocele aspiration is considered positive if the volume aspirated exceeds 120cc.
  - The number of repeated lymphocele aspirations and the volume.
  - Adjuvant chemotherapy starting date, if required
  - Recording of adverse events:
- Follow-up visits at two months (V6)
  - Recording of adverse events:

### 3.5.3. Evaluation criteria

#### 3.5.3.1. Endpoints

**Primary endpoint**

The primary endpoint of this study is the ratio of patients who did not have symptomatic, postoperative axillary lymphoceles defined as the absence of aspiration or a unique or iterative aspirations global volume of lymphoceles inferior to 60cc inclusive (≤) in the 28 days after the intervention or a systematic aspiration volume at the 28^th^ day inferior to 120cc inclusive (≤)

**Secondary endpoints**

The secondary objectives of this study are to evaluate:

- the duration of postoperative drainage
- the daily drainage volume
- the total drainage volume
- the number of repeated lymphocele aspirations and the volume.
- the total volume of lymph aspirated
- the incidence of postoperative febrile episodes
- the length of the hospital stay
- the length of time to onset of adjuvant chemotherapy

**3.5.4 Evaluation of medicinal dosages and of pharmacokinetic**

A pharmacokinetic study will be realized with a sample before injection at V1 (injection day) and a sample at V4 (J_19-23_ post-injection). If the V4 visit corresponds to more than 24 days after injection, this visit will be advanced in order to respect samples times. An electrocardiogram (ECG) will be done at the same time that sampling for pharmacokinetic.

# 6. Statistical methods

## 6.1. Statistical methods to be used

We recommend a prospective, randomized, two-center trial according to a Bayesian design with a control arm receiving a placebo.

## 6.2. Efficacy evaluation:

The evaluation criterion will be the proportion of patients who did not have symptomatic, postoperative axillary lymphoceles defined as the absence of aspiration or a unique or iterative aspirations global volume of lymphoceles inferior to 60cc inclusive (≤) in the 28 days after the intervention or a systematic aspiration volume at the 28th day inferior to 120cc inclusive (≤).

## 6.3. Safety assessment

Safety will be evaluated during each visit and recorded in the CRFs.

## 6.4. Number of subjects

A total of 90 patients will be included over 12 months with 45 patients in the treatment group and 45 patients in the treatment-free group. In principle, there is no need to justify the number of subjects in a Bayesian study (15). However, the concept of desired accuracy of the success rate can be used based on its distribution a posteriori to define a test sample to be included.

## 6.5. Primary endpoint analysis

The statistical analysis will be carried out sequentially after observing the principal criterion (i.e. success is defined as a total volume of lymphoceles following single or repeated aspiration ≤ 60 cc in the 28 days following surgery or a routine aspiration volume on the 28^th^ day ≤ 120cc) of each patient included for each randomization group, with or without treatment.

It involves estimating the probability of a response in each group (proportion of patients who will not have undergone lymphocele aspiration or the aspiration of a total volume of ≤ 60cc or ≤ 120cc for the aspiration carried out on the 28^th^ day after surgery) using a Bayesian design based on a beta-binomial model (16,17). With the Bayesian approach, the response rate in each group (π*_i_*) is considered as a random variable *primarily* of density focused on the anticipated response rate of 80% in the group receiving treatment and 60% in the treatment-free group, which will be sequentially updated as the observations are made according to a so-called *a posteriori* law.

*A priori* density will be selected for each group from the Beta conjugated laws group, defined by its two parameters *a_i_*and *b_i_* (with a life expectancy = and variance = , with *i=1,2* according to the group considered). The Bayesian estimator of the response rate in each group will be expectancy of the *a posteriori* law, i.e. the Beta law of parameters *A_ni_* and *B_ni_* , defined on the basis of those of the *a priori* law and the number *n_i_* of inclusions, as shown below. *A_ni_=a+r_i_* and *B_ni_=b+n_i_-r_i_,* where *r_i_* represents the number of responses observed on the *n_i_* included in the *i* group. The following estimator is, therefore, obtained: .

## 6. 6. Rules for discontinuation

Bayesian rules for discontinuation will be calculated sequentially in order to possibly make a decision to stop inclusions in eac group based on the four following criteria (17, 18):

- - The first two discontinuation criteria are defined on the basis of the *a posteriori* law of *π_ι_*: (i) ***criterion 1*** = *a posteriori* probability that the response rate *π_ι_ *is less than 60% (considered as the maximum inefficacy threshold) and (ii) ***criterion 2*** = *a posteriori* probability that the response rate *π_ι_ *is greater than 80% (considered as the minimum efficacy threshold) will be calculated*.*
  - The third discontinuation criterion will be based on the predictive distribution of responses (*s*) out of *z_i_=5* next included , i.e., ***criterion 3*** = $P(r_{i}>3\left|z_{i}=5,data\right.)$
  - The fourth discontinuation criterion will be based on the maximum expected gain in terms of the width of the 95% credibility interval of the response rate out of z = 5 next included: ***criterion 4*** = $\max_{(y_{1i},...,y_{zi})} \left|c_{\alpha,ji+zi}(\pi_{i})-c_{\alpha,ji+1}(\pi_{i})\right|$, where$c_{\alpha,j+1}(\pi)$is the 95% credibility interval (α= 0.05) of π, calculated after inclusion of *j_i_* subjects, such that $\int_{\pi_{\max}}^{\pi_{\min}} f(\theta\left|\Omega_{ji}\right.)d\theta=1-\alpha$.

The discontinuation decisions are as follows:

- - - - *If criterion 1 > 0.90: discontinuation of inclusions with conclusion of inefficacy*
      - *If criterion 2 > 0.90, discontinuation of inclusions with conclusion of efficacy*
      - *If criterion 3 > 0.90: discontinuation of inclusions with conclusion of efficacy*
      - *If criterion 4 < 0.05: discontinuation of inclusions with conclusion of efficacy*

An independent committee will meet to confirm the decision to continue or discontinue inclusions in the considered group based on the results of the sequential analysis.

# 8. References

1. Harris AG. Future medical prospects for Sandostatin. Metabolism 1990;39(2):180–185

2. Ulibarri JI, Sanz Y, Fuentes C, et al. Reduction of lymphorrhagia from ruptured thoracic duct by somatostatin. Lancet 1990;336:258.

3. Schmid et al AACE 2005 présentation

3. Carcoforo P, Soliani G, Maestroni U, Donini A, Inderbitzin D, Hui TT, Lefor A, Avital I, Navarra G. Octreotide in the treatment of lymphorrhea after axillary node dissection: a prospective randomized controlled trial. J Am Coll Surg. 2003 Mar;196(3):365-9.

4. Mahmoud SA, Abdel-Elah K, Eldesoky AH, El-Awady SI. Octreotide can control lymphorrhea after axillary node dissection in mastectomy operations. Breast J. 2007 Jan-Feb;13(1):108-9.

5. Capocasale E, Busi N, Valle RD, Mazzoni MP, Bignardi L, Maggiore U, Buzio C, Sianesi M. Octreotide in the treatment of lymphorrhea after renal transplantation: a preliminary experience. Transplant Proc. 2006 May;38(4):1047-8.

7. Schmid HA, Schoeffter P. Functional activity of the multiligand analog SOM230 at human recombinant somatostatin receptor subtypes supports its usefulness in neuroendocrine tumors. Neuroendocrinology. 2004;80 Suppl 1:47-50.

8. Bruns C, Lewis I, Briner U, Meno-Tetang G, Weckbecker G. SOM230: a novel somatostatin peptidomimetic with broad somatotropin release inhibiting factor (SRIF) receptor binding and a unique antisecretory profile. Eur J Endocrinol. 2002 May;146(5):707-16.

9. Matulonis UA, Seiden MV, Roche M, Krasner C, Fuller AF, Atkinson T, Kornblith A, Penson R. Long-acting octreotide for the treatment and symptomatic relief of bowel obstruction in advanced ovarian cancer. J Pain Symptom Manage. 2005 Dec;30(6):563-9.

10. Mangili G, Franchi M, Mariani A, Zanaboni F, Rabaiotti E, Frigerio L, Bolis PF, Ferrari A. Octreotide in the management of bowel obstruction in terminal ovarian cancer. Gynecol Oncol. 1996 Jun;61(3):345-8.

11. Massacesi C, Galeazzi G. Sustained release octreotide may have a role in the treatment of malignant bowel obstruction. Palliat Med. 2006 Oct;20(7):715-6.

12. Mystakidou K, Tsilika E, Kalaidopoulou O, Chondros K, Georgaki S, Papadimitriou L. Comparison of octreotide administration vs conservative treatment in the management of inoperable bowel obstruction in patients with far advanced cancer: a randomized, double- blind, controlled clinical trial. Anticancer Res. 2002 Mar-Apr;22(2B):1187-92.

13.http://www.afssaps.fr/Infos-de-securite/Recommandations-de-bonne-pratique/Soins-palliatifs-specificite-d-utilisation-des-medicaments-courants-hors antalgiques.fr

14. Hanfelt JJ, Slack RS, Gehan EA. A modification of Simon's optimal design for phase II trials when the criterion is median sample size. Control Clin Trials. 1999 Dec;20(6):555-66.

15. Spiegelhalter D.J., Abrams K.R., Myles J.P. (2004) Bayesian approaches to clinical trials and health-care evaluation. John Wiley & Sons Ltd., Chischester, England, 2004.

16. Berry D. A. Monitoring accumulating data in a clinical trial (1989) Biometrics 45, 1197-211

17. Zohar S, Teramukai S, Zhou Y. Bayesian design and conduct of phase II single-arm clinical trials with binary outcomes: a tutorial. (2008) Contemp Clin Trials 29(4):608-16.

18. Zohar, S. and Chevret, S. The continual reassessment method: Comparison of bayesian stopping rules for dose-ranging studies. (2001) Stat Med, 20: 2827-2843
