# Supplementary material for: Evaluation of the Effects of Pasireotide LAR Administration on Lymphocele Prevention after Axillary Node Dissection for Breast Cancer: Results of a Randomized Non-Comparative Phase 2 Study
Source: PLoS One. 2016 Jun 9;11(6):e0156096. doi: 10.1371/journal.pone.0156096 (PMC4900597; doi:10.1371/journal.pone.0156096)
Supplement: S3 File — (DOC) [file pone.0156096.s003.doc]

**ÉVALUATION DE L’INTERET DE L’ADMINISTRATION D’UN ANALOGUE DE L’OCTREOTIDE (SOM 230®) DANS LA PREVENTION DES LYMPHOCELES APRES CURAGE AXILLAIRE**

**ESSAI PROSPECTIF RANDOMISE PHASE 2**

**Promoteurs :**

**Alliance pour la recherche en Cancérologie (APREC), Hôpital Tenon**

**Investigateurs coordonateurs :**

**Pr Roman Rouzier, Dr C Mazouni, Dr Catherine Uzan, Dr Elisabeth Chéreau**

**Investigateurs associés :**

**Pr S. Uzan, Dr F. Rimareix, Pr E. Daraï, Dr M. Ballester, Dr S. Gouy, Dr JR. Garbay**

**- Service de Gynécologie Obstétrique et Reproduction Humaine, Pr. S. Uzan**

**Hôpital Tenon, AP-HP**

**- Service de Chirurgie, Dr D. Elias**

**Institut Gustave Roussy**

**Soutien : Novartis**

**Index**

1. **OBJECTIFS**
2. **TYPE D'ÉTUDE**
3. **RATIONNEL**
4. **CALCUL DE PUISSANCE ET RECRUTEMENT**
5. **CRITÈRES D'INCLUSION, D'EXCLUSION ET D'ARRÊT DE L'ETUDE**
6. **CRITÈRES DE JUGEMENT**
7. **DÉROULEMENT DE L'ÉTUDE**
8. EVALUATION DE LA SECURITE
9. STATISTIQUES
10. DROIT D'ACCES AUX DONNEES ET DOCUMENTS SOURCE
11. CONSIDERATIONS LEGALES ET ETHIQUES
12. TRAITEMENT DES DONNEES ET CONSERVATION DES DOCUMENTS ET DES DONNEES RELATIVES A LA RECHERCHE
13. **CONSIDERATIONS PHARMACO-ECONOMIQUES**
14. ASSURANCE ET ENGAGEMENT SCIENTIFIQUE
15. **BIBLIOGRAPHIE**

**ANNEXE 1. FICHE D'INFORMATION ET CONSENTEMENT ÉCLAIRÉ**

**ANNEXE 2. MODELE DE TIMMERMAN**

**SYNOPSIS**

1. **Contexte**

La principale morbidité après curage axillaire dans le cadre d’une chirurgie carcinologique du sein est la survenue post opératoire d’un lymphocèle. Dans la littérature, ce taux peut varier de 4 à 89 % en fonction du type de chirurgie, de la mise en place ou non d’un drainage ou d’un pansement compressif, du moment d’ablation du drain… Dans notre expérience, l’incidence est de 40% (données IGR portant sur 70 patientes de novembre 2008 à février 2009).

Deux études récentes rapportent des résultats encourageants sur la diminution des lymphocèles post opératoires, de la durée et du débit des drains avec l’utilisation d’Octréotide. Une nouvelle molécule développée par le laboratoire Novartis, la pasireotide (SOM 230®) est un analogue de la somatostatine possédant une forte affinité pour quatre des cinq récepteurs à la somatostatine.

1. **Objectif**

L’objectif de cet essai est d’évaluer l’incidence post opératoire des lymphocèles après curage axillaire avec injection post opératoire de SOM 230®.

1. **Design de l’essai**

Nous proposons un essai prospectif, bicentrique, de phase 2 à deux étapes avec un bras contrôle randomisé.

1. **Population de l’essai**

Toutes les patientes pour lesquelles une indication de mastectomie - curage axillaire est posé en pré-opératoire seront éligibles. Population témoin de taille identique : randomisation 1:1.

1. **Déroulement de l’essai**

- Critères d’inclusion : toute patiente ayant une chirurgie mammaire programmée avec indication pré opératoire de mastectomie curage axillaire.

- Critères d’exclusion : patiente mineure ou ne comprenant pas le Français, refus de la patiente, procédure du ganglion sentinelle programmée, contre-indication à l’administration d’Octréotide (grossesse, allaitement, allergie).

- Inclusion et signature du consentement pré opératoire.

- 1 injection de SOM 230® LP 15 à 10 jours avant l’intervention en intra musculaire

- Recueil des données opératoires et post opératoires (apparition d’une lymphocèle, nécessité de ponction, nombre et volume de chaque ponction itérative, débit quotidien des drains, durée de drainage, complications locales infectieuses ou retard de cicatrisation).

1. **Objectifs primaire et secondaires**

- Objectif primaire : réduction de l’incidence des lymphocèles post opératoires nécessitant une ou des ponctions itératives

- Objectifs secondaires : quantité totale de lymphe drainée à J5 et jusqu’à ablation des drains, durée de drainage, débit quotidien des drains, cicatrisation locale, infections, fièvre, durée d’hospitalisation, délai de démarrage de la chimiothérapie adjuvante.

1. **Analyse statistique et plan de l’essai**

Le but est de montrer une réduction de l’incidence des lymphocèles de 40% à 20%. Pour ceci, la première étape doit inclure 25 patientes. L’essai sera arrêté à l’issue de la première étape si plus de 13 évènements sont enregistrés et la SOM 230® sera considérée comme inefficace. Si seulement 4 lymphocèles sont observées, l’essai sera arrêté et la SOM 230® sera considérée comme efficace. La seconde étape nécessitera l’inclusion de 20 patientes supplémentaires. À la fin de l’essai, l’effet bénéfique de l’injection de SOM 230® LP sera démontré si 13 patientes ne présentent pas de lymphocèle post opératoire. 45 patientes seront donc nécessaires pour une puissance de 80% avec un risque alpha à 0,05.

Un groupe témoin de 45 patientes sera inclus en parallèle par randomisation afin de valider l’incidence des lymphocèles post-opératoire.

Le nombre total de patientes enrôlées sera donc de 90 patientes (45 traitées – 45 témoins).

**INTRODUCTION**

L’Octréotide, un analogue de la somatostatine, a montré son efficacité dans la prise en charge médicale des fistules digestives et pancréatiques post opératoires. Deux études récentes ont montré son intérêt dans la réduction des lymphocèles après curage axillaire dans le cadre de la chirurgie du cancer du sein.

La principale morbidité du curage axillaire dans le cadre de la chirurgie cancérologique mammaire est l’apparition post opératoire après l’ablation du drain axillaire d’une lymphocèle. Celle-ci peut être source de douleurs, de ponctions itératives, d’infection et de retard à la cicatrisation locale.

La pasireotide, SOM 230®, analogue de la somatostatine en cours d’évaluation, possédant une plus forte affinité (30 à 40 fois supérieure) pour 4 des 5 récepteurs à la somatostatine, est une molécule attractive dans cette indication.

À partir des résultats encourageants publiés avec l’octréotide et des effets attendus supérieurs avec la pasireotide, nous souhaitons mettre en évidence un réel bénéfice de l’administration postopératoire de SOM 230® SC dans la diminution de l’incidence des lymphocèles axillaires après une mastectomie – curage axillaire.

**1. OBJECTIFS**

L’objectif principal de cette étude est de mettre en évidence une réduction de l’incidence des lymphocèles axillaires post-opératoire, grâce à l’administration systématique préopératoire de SOM 230®.

Les objectifs secondaires de cette étude sont d’évaluer :

- la durée de drainage post opératoire
- le débit quotidien des drains
- le débit total des drains
- l’incidence des épisodes fébriles post opératoires
- la durée d’hospitalisation
- le nombre de ponctions itératives de lymphocèle et le volume
- le délai de démarrage de la chimiothérapie adjuvante

**2. TYPE D'ETUDE**

Étude bicentrique, prospective, de phase 2 en deux étapes avec un groupe contrôle randomisé.

Schéma de l'étude:

- Signature du consentement lors de l’inclusion (en pré opératoire)
- Recueil des données opératoires (type d’intervention, durée, complications per opératoires, type et nombre de drains)
- Recueil des données post opératoires :
  - Débit quotidien des drains
  - Durée totale de drainage
  - Complications post-opératoires (hématome, nécrose cutanée…)
  - Nécessité de ponctionner une lymphocèle symptomatique
  - Durée d’hospitalisation
  - Nombre et volume des ponctions itératives de lymphocèle
  - Date de début de la chimiothérapie adjuvante

**3. BASE RATIONNELLE**

**3.1 MODE D’ACTION DE LA PASIREOTIDE**

La somatostatine est une hormone largement distribuée par le système nerveux et gastro-pancréatique responsable de divers effets pharmacologiques et physiologiques. Elle peut inhiber la sécrétion endocrine et exocrine gastro-intestinale et elle possède une action anti-inflammatoire (1). L’effet direct de la somatosatine sur le flux lymphatique a été observé uniquement sur le tractus gastro-intestinal.

Plusieurs séries rapportent l’utilisation de l’octréotide dans le traitement de l’ascite chyleuse ou dans la prise en charge des plaies du canal thoracique (2). Même si son mécanisme d’action n’est pas bien étudié, il est probable qu’elle agisse en inhibant le flux sanguin splanchnique et en limitant l’absorption des triglycérides.

Des récepteurs à la somatostatine ont été mis en évidence dans les tissus lymphatiques y compris en dehors du tractus intestinal. Il est donc probable que l’action inhibitrice de la somatostatine sur le flux lymphatique gastro-intestinal puisse s’étendre en dehors du tractus intestinal et notamment sur le système lymphatique.

La somatostatine pourrait donc permettre de réduire le flux lymphatique après lymphadénectomie notamment axillaire.

Une nouvelle molécule, la pasireotide (SOM 230®) est actuellement en cours de développement. Cette molécule est un analogue de la somatostatine et possède une affinité supérieure à l’octréotide pour quatre des cinq récepteurs à la somatostatine (sst1, sst2, sst3 et sst5). Elle aurait donc un effet supérieur à l’octréotide.

La forme à libération progressive nécessite une injection préopératoire intra musculaire. La concentration efficace en plateau est obtenue au bout de 10 jours quelque soit le dosage choisi (20,40 ou 60 mg) (3)

**3.2 REVUE DE LA LITTERATURE**

Hormis les nombreux articles de la littérature détaillant les effets bénéfiques de l’octréotide sur les fistules digestives et pancréatiques et sur la régression des ascites chyleuses et chylothorax, quelques rares articles ont cherché à mettre en évidence son effet sur les lymphocèles post-opératoires.

D’une part, deux articles mettent en évidence l’effet positif de la somatostatine dans la réduction du débit des drains et de l’incidence des lymphocèles après curage axillaire dans le cancer du sein :

- Carcoforo et al (4) en 2003 ont mis en évidence une différence significative entre deux groupe recevant ou non en post opératoire immédiat de l’Octréotide en sous cutané. En effet, dans le groupe traité, il a été noté une diminution du débit moyen des drains (65,4 vs 94,6 ml, p=0,0001) et une ablation plus précoce des drains en post opératoire (7,1 vs 16,7 jours, p=0,0001).
- Un autre article plus récent de Mahmoud et al (5) a montré les mêmes effets bénéfiques sur le débit moyen journalier des drains (104 vs 145 ml, p=0,0001), sur la durée total de drainage (12,7 vs 25 jours, p=0,0001) et sur la nécessité de ponction des lymphocèles post opératoire (90 vs 40%, p=0,0001).

Dans un autre domaine, celui de la transplantation rénale, un article de Capocasale et al (6) en 2006 montre une réduction de la durée d’écoulement lymphatique post transplantation dans le groupe ayant reçu de l’Octréotide. De même, il met en évidence une diminution de l’apparition de lymphocèle après retrait des drainages.

La pasireotide est un analogue injectable de la somatostatine. Comme la somatostatine naturelle et les analogues connus, son efficacité pharmaceutique dépend de sa liaison aux récepteurs de la somatostatine. Ceux-ci sont au nombre de cinq (sst 1 à 5) et sont exprimés dans les différents tissus de l’organisme dans les conditions physiologiques normales. Les analogues de la somatostatine activent ces récepteurs ce qui entraîne une diminution de l’activité cellulaire et une inhibition de la synthèse hormonale. (7). L’octréotide et la lanreotide utilisés actuellement possèdent une forte affinité pour le récepteur sst2 et une affinité modérée voire absente pour les autres types. La pasiréotide possède une affinité bien supérieure à l’octréotide pour certains des récepteurs de la somatostatine : 30 fois pour sst1, 5 fois pour sst3, 40 fois pour sst5. Elle est équivalente pour le sst2 et absence (comme pour l’octréotide) pour le sst4. (8)

Les effets secondaires décrits avec la pasireotide sont la possibilité d’une hyperglycémie post prandiale transitoire dose dépendante et apparaît essentiellement à partir d’injections à 600μg. Des épisodes de troubles intestinaux ont été rapportés (diarrhée, nausées, vomissements) mais ne nécessitant généralement pas de traitement médicamenteux et disparaissant spontanément au cours du traitement.

Dans la littérature, l’incidence des lymphocèles axillaires après chirurgie cancérologique mammaire est très variable : une méta analyse récente reprenant 66 études retrouve des taux allant de 4 à 89 % selon la présence ou non d’un drainage, le type de chirurgie (conservatrice ou non), le moment d’ablation du drain, la mise en place ou non d’un pansement compressif.

Dans notre expérience, l’incidence à l’hôpital Tenon est de 40%. Ce pourcentage a été confirmé par les données de l’Institut Gustave Roussy qui retrouve un taux de 39,3% sur 70 patientes opérées entre novembre 2008 et février 2009.

**3.3 ANALOGUES DE LA SOMATOSATINE ET CANCER**

Plusieurs équipes ont rapporté leur utilisation de la Sandostatine® ou d’autres analogues de la somatostatine dans la prise en charge des patients atteints de carcinose péritonéale non opérable (9-12). Ce traitement était alors utilisé comme un soin de support et permettait de réduire l’incidence des symptômes liés au syndrome obstructif. En effet il permet de réduire les sécrétions digestives. Certains auteurs ont même suggéré un effet anti-tumoral des analogues de la somatostatine (9).

**4. CALCUL DE PUISSANCE ET RECRUTEMENT**

Pour cette étude de phase II, nous réaliserons une étude selon un plan à 2 étapes :

- hypothèse nulle (H0) : l’incidence des lymphocèles post opératoire est de 40%

- hypothèse alternative (H1) : l’administration systématique de SOM 230® permet de réduire l’incidence des lymphocèles post opératoire à 20%

- une erreur de type I à 5%, et une puissance de 80%

Inclusion de 25 patientes pour la première étape avec rejet de l’hypothèse alternative s’il existe plus de 13 lymphocèles et acceptation de H1 si il existe moins de 4 lymphocèles.

Inclusion de 20 patientes supplémentaires pour la seconde étape avec effet bénéfique démontré (on accepte H1) si 13 patientes ne présentent pas de lymphocèle symptomatique post opératoire.

Un groupe témoin de 45 patientes sera randomisé en parallèle afin de valider l’incidence des lymphocèles post opératoires.

90 patientes seront donc enrôlées au total.

**5. CRITERES D'INCLUSION ET D'EXCLUSION ET D'ARRÊT DE L'ETUDE**

**Critères d'inclusion**

- Patiente majeure.
- Patiente comprenant le Français.
- Patiente ayant une prise en charge par la sécurité sociale.
- Patiente ayant une chirurgie mammaire programmée avec indication pré opératoire de mastectomie curage axillaire

**Critères d'exclusion**

- Patiente mineure.
- Patiente ne comprenant pas le Français.
- Patiente n’ayant pas de prise en charge par la sécurité sociale.
- Patiente présentant une ou des contre-indications à l'anesthésie et à la chirurgie.
- Patiente présentant une contre-indication à l’administration de Pasireotide
- Diabète connu
- Refus de la patiente
- Procédure du ganglion sentinelle programmée

**Critère d'arrêt de l'étude**

- Plus de 13 lymphocèles au cours de la première étape de l’étude

**6. CRITERES DE JUGEMENT**

**Critère principal de jugement**

Incidence des lymphocèles post opératoires symptomatiques nécessitant une ou des ponctions itératives.

**Critères secondaires de jugement**

- Débit journalier de chaque drain
- Durée totale de drainage
- Cicatrisation locale
- Infection locale
- Incidence des épisodes fébriles sans point d’appel clinique identifié
- Durée totale d’hospitalisation
- Délai de démarrage de la chimiothérapie adjuvante

**7. DEROULEMENT DE L'ETUDE**

**7.1 Pré opératoire**

Signature du consentement par la patiente

Recueil des antécédents et notamment la présence d’un diabète, d’une obésité (calcul du BMI) et l’administration d’une chimiothérapie néo-adjuvante.

Une injection intra-musculaire de SOM 230® 5 à 10 jours avant l’intervention.

**7.2 Déroulement opératoire**

Recueil des données opératoires

- Durée
- Complications per opératoires
- Nombre et localisation des drains
- Type et calibre des drains
- Mise en place ou non d’un pansement compressif

Le nombre et la position des drains sont laissés à l’appréciation de l’opérateur.

**7.3 Période post opératoire**

Surveillance post opératoire :

- Complications
- Débit journalier des drains
- Durée totale de drainage
- Incidence des infections locales et de la fièvre
- Durée d’hospitalisation
- Délai de démarrage de la chimiothérapie adjuvante

Il est décidé l’ablation des drains à partir d’un débit journalier inférieur à 50 cc et une durée totale de drainage limitée à 7 jours. Le vide est retiré du drain à partir du 5^ème^ jour post opératoire.

Déclaration systématique des éléments indésirables graves auprès de la pharmacovigilance.

**7.4 Durée de l’étude – Délais**

Début de l’inclusion des patientes : Janvier 2010

Fin de l’inclusion : Juin 2011

Analyse des données terminées : Décembre 2011

8. EVALUATION DE LA SECURITE

**8.1. Description des paramètres d'évaluation de la sécurité**

- **Evènement indésirable**

Toute manifestation nocive survenant chez une personne qui se prête à une recherche biomédicale que cette manifestation soit liée ou non à tout élément expérimental de la recherche et ce qu’il s’agisse des actes pratiqués ou des produits utilisés.

- **Effet indésirable**

Toute réaction nocive et non désirée à tout élément expérimental de la recherche et ce qu’il s’agisse des actes pratiqués ou des produits utilisés.

- **Evènement ou effet indésirable grave**

Tout évènement ou effet indésirable qui entraîne la mort, met en danger la vie de la personne qui se prête à la recherche, nécessite une hospitalisation ou la prolongation de l’hospitalisation, provoque une incapacité ou un handicap importants ou durables, ou bien se traduit par une anomalie ou une malformation congénitale.

- **Effet indésirable inattendu**

Tout effet indésirable dont la nature, la sévérité ou l’évolution ne concorde pas avec les informations figurant dans les référentiels reconnus par les autorités.

- **Fait nouveau**

Toute nouvelle donnée de sécurité, pouvant conduire à une réévaluation du rapport des bénéfices et des risques de la recherche ou qui pourrait être suffisant pour envisager des modifications des documents relatifs à la recherche, de la conduite de la recherche ainsi que, le cas échéant, dans l’utilisation du produit.

## 8.2. Méthodes et calendrier prévus pour mesurer, recueillir et analyser les paramètres d'évaluation de la sécurité

8.2.1. Comité de pilotage

Il sera constitué des initiateurs cliniciens du projet, du biostatisticien en charge du projet, et des représentants du promoteur nommés pour cette recherche.

Il définira l'organisation générale et le déroulement de la recherche et coordonnera les informations.

Il déterminera décidera en cours de recherche des conduites à tenir dans les cas imprévus, surveillera le déroulement de la recherche en particulier sur le plan de la tolérance et des évènements indésirables.

### 8.2.2. Comité de surveillance indépendant

Il a une fonction consultative et décisionnelle lorsque le promoteur fait appel à lui sur des points médicaux tels la tolérance et les événements indésirables. il est constitué de personnes extérieures à la recherche dont nécessairement un clinicien spécialiste de la pathologie étudiée et un pharmacologue/pharmacovigilant et selon le protocole un méthodologiste/biostatisticien. Les modalités de fonctionnement seront précisées ultérieurement.

## 8.3. Procédures mises en place en vue de l'enregistrement et de la notification des évènements indésirables

**8.3.1. Evènements indésirables non graves :**

Tout événement indésirable - non grave suivant la définition précédente - observé lors de la recherche et dans ses suites devra être reporté dans le cahier d’observation dans la section prévue à cet effet.

Un seul évènement doit être reporté par item. L’évènement peut correspondre à un symptôme, un diagnostic ou à un résultat d’examen complémentaire jugé significatif. Tous les éléments cliniques ou para-cliniques permettant de décrire au mieux l’évènement correspondant doivent être reportés.

**8.3.2. Evènements indésirables graves (EIG) :**

Le formulaire de déclaration d’un événement indésirable grave, validé pour la recherche, est inclus dans le protocole en annexe. Il en est de même pour la grille de classification des événements indésirables graves et non graves. Cette grille a été mise en place afin d’aider l’investigateur dans sa gestion des événements indésirables (c’est-à-dire l’aider à différencier les événements selon leur gravité et leur caractère attendu). La grille est élaborée et validée par l’ensemble des acteurs impliqués dans la recherche. Elle peut être amenée à évoluer, au décours de la recherche, en fonction des déclarations reçues par le promoteur.

L’investigateur est tenu de notifier immédiatement au promoteur (APREC) tous les événements indésirables graves à l’exception de ceux recensés dans la grille comme ne nécessitant pas une notification immédiate.

L'investigateur complète le formulaire de déclaration d’évènement indésirable grave (du cahier d'observation de la recherche) et l’envoie au DRCD par fax au 01 44 84 17 99 et ce, dans les 48 heures (après si possible un appel téléphonique immédiat au 01 44 84 17 23 en cas de décès ou d’une menace vitale inattendus).

L’investigateur doit également informer le promoteur en charge de la recherche de la survenue de l’EIG.

Pour chaque évènement indésirable grave, l’investigateur devra émettre un avis sur le lien de causalité de l’évènement avec tout élément expérimental de la recherche et ce qu’il s’agisse des actes pratiqués ou des produits utilisés.

L’obtention d’informations relatives à la description et l’évaluation d’un évènement indésirable peuvent ne pas être possibles dans le temps imparti pour la déclaration initiale.

Aussi, l'évolution clinique ainsi que les résultats des éventuels bilans cliniques et des examens diagnostiques et/ou de laboratoire, ou toute autre information permettant une analyse adéquate du lien de causalité seront rapportés :

soit sur la déclaration initiale d’EIG s’ils sont immédiatement disponibles,

soit ultérieurement et le plus rapidement possible, en envoyant par fax une nouvelle déclaration d’EIG complétée (et en précisant qu’il s’agit d’un suivi d’EIG déclaré et le numéro de suivi).

Toutes les déclarations faites par les investigateurs devront identifier chaque sujet participant à la recherche par un numéro de code unique attribué à chacun d’entre eux.

En cas de décès notifié d’un sujet participant à la recherche, l’investigateur communiquera au promoteur tous les renseignements complémentaires demandés (compte-rendu d’hospitalisation, résultats d’autopsie…).

Tout fait nouveau survenu dans la recherche ou dans le contexte de la recherche, provenant de données de la littérature ou de recherches en cours, devra être notifié au promoteur.

- Déclaration des évènements indésirables graves aux Autorités de Santé

Elle sera assurée par le Pôle de Pharmacovigilance du DRCD, après évaluation de la gravité de l’évènement indésirable, du lien de causalité avec l’élément expérimental de la recherche et ce qu’il s’agisse des actes pratiqués ou des produits utilisés , ainsi que du caractère inattendu des effets indésirables.

Toutes les suspicions d’effet indésirable grave inattendu seront déclarées par le promoteur aux autorités compétentes dans les délais légaux.

En cas d’effet indésirable grave inattendu dû à un élément expérimental de la recherche et ce qu’il s’agisse des actes pratiqués ou des produits utilisés, les autorités compétentes, le Comité de Protection des Personnes et les investigateurs de la recherche devront être informés.

## 8.4. Modalités et durée du suivi des personnes suite à la survenue d'évènements indésirables

Tout patient présentant un évènement indésirable doit être suivi jusqu’à la résolution ou la stabilisation de celui-ci.

1. Si l’évènement n’est pas grave, l’évolution en sera notée sur la page correspondante du cahier d’observation à la section prévue à cet effet.
2. Si l’évènement est grave, un suivi d’EIG sera envoyé au DRCD.

9. STATISTIQUES

Un cahier d’observation sera préparé par le promoteur comportant les différentes informations nécessaires à l’évaluation. Le remplissage des données sera réalisé par le technicien de recherche clinique, recruté par le promoteur, sous la responsabilité de l'investigateur.

La saisie sera réalisée par le TEC sur un masque ACCESS conçu par le promoteur.

Un data management des données sera réalisée au fur et à mesure de la saisie par le datamanager de le promoteur.

L’analyse statistique sera réalisée par un prestataire indépendant.

10. DROIT D'ACCES AUX DONNEES ET DOCUMENTS SOURCE

Les personnes ayant un accès direct conformément aux dispositions législatives et réglementaires en vigueur, notamment les articles L.1121-3 et R.5121-13 du code de la santé publique (par exemple, les investigateurs, les personnes chargées du contrôle de qualité, les moniteurs, les assistants de recherche clinique, les auditeurs et toutes personnes appelées à collaborer aux essais) prennent toutes les précautions nécessaires en vue d'assurer la confidentialité des informations relatives aux médicaments expérimentaux, aux essais, aux personnes qui s'y prêtent et notamment en ce qui concerne leur identité ainsi qu’aux résultats obtenus. Les données collectées par ces personnes au cours des contrôles de qualité ou des audits sont alors rendues anonymes.

10.1. Contrôle et assurance de la qualité

La recherche sera encadrée selon les procédures opératoires standard du promoteur.

Le déroulement de la recherche dans les centres investigateurs et la prise en charge des sujets seront faites conformément à la déclaration d’Helsinki et les Bonnes Pratiques en vigueur.

**10.2. Procédures de monitoring**

Risque évalué de la recherche : **B**

Les ARC représentants du promoteur effectueront des visites des centres investigateurs au rythme correspondant au schéma de suivi des patients dans le protocole, aux inclusions dans les différents centres et au niveau de risque qui a été attribué à la recherche.

- Visite d‘ouverture de chaque centre : avant inclusion, pour une mise en place du protocole et prise de connaissance avec les différents intervenants de la recherche biomédicale.

- Lors des visites suivantes, les cahiers d'observation seront revus au fur et à mesure de l'état d'avancement de la recherche par les ARC. L'investigateur principal de chaque centre ainsi que les autres investigateurs qui incluent ou assurent le suivi des personnes participant à la recherche s’engagent à recevoir les ARC à intervalles réguliers.

Lors de ces visites sur site et en accord avec les Bonnes Pratiques Cliniques, les éléments suivants seront revus :

- Respect du protocole et des procédures définies pour la recherche,
- Vérification des consentements éclairés des patients
- Examen des documents source et confrontation avec les données reportées dans le cahier d’observation quant à l’exactitude, les données manquantes, la cohérence des données.

- Visite de fermeture : récupération des cahiers d’observation, bilan à la pharmacie, documents de la recherche biomédicale, archivage.

### 10.3. Transcription des données dans le cahier d’observation

Toutes les informations requises par le protocole doivent être fournies dans le cahier d’observation et une explication donnée par l’investigateur pour chaque donnée manquante.

Les données devront être transférées dans les cahiers d'observation au fur et à mesure qu'elles sont obtenues qu'il s'agisse de données cliniques ou para-cliniques. Les données devront être copiées de façon nette et lisible à l'encre noire dans ces cahiers (ceci afin de faciliter la duplication et la saisie informatique).

Les données erronées dépistées sur les cahiers d'observation seront clairement barrées et les nouvelles données seront copiées sur le cahier avec les initiales et la date par le membre de l'équipe de l'investigateur qui aura fait la correction.

L'anonymat des sujets sera assuré par un numéro de code et les initiales de la personne qui se prête à la recherche sur tous les documents nécessaires à la recherche, ou par effacement par les moyens appropriés des données nominatives sur les copies des documents source, destinés à la documentation de la recherche.

Les données informatisées sur un fichier seront déclarées à la CNIL selon la procédure adaptée au cas.

11. CONSIDERATIONS LEGALES ET ETHIQUES

Le promoteur est défini par la loi 2004-806 du 9 août 2004. Dans cette recherche, l’**Alliance pour la recherche en Cancérologie (APREC)** est le promoteur et le Département de la Recherche Clinique et du Développement (DRCD) en assure les missions réglementaires.

Avant de démarrer la recherche, chaque investigateur fournira au représentant du promoteur de la recherche une copie de son curriculum vitæ personnel daté et signé, comportant son numéro d’inscription à l’ordre des médecins et son numéro ADELI

11.1. Demande d’autorisation auprès du ministère de la santé (DGS)

Pour pouvoir démarrer la recherche, l’AP-HP en tant que promoteur doit soumettre un dossier de demande d’autorisation auprès de l'autorité compétente la DGS. L'autorité compétente, définie à l'article L. 1XIV3-XIV, se prononce au regard de la sécurité des personnes qui se prêtent à une recherche biomédicale, en considérant notamment la sécurité et la qualité des produits utilisés au cours de la recherche conformément, le cas échéant, aux référentiels en vigueur, leur condition d'utilisation et la sécurité des personnes au regard des actes pratiqués et des méthodes utilisées ainsi que les modalités prévues pour le suivi des personnes.

11.2. Demande d’avis au Comité de Protection des Personnes

En accord avec l'article L.1XIV3-6 du Code de Santé Publique, le protocole de recherche doit être soumis par le promoteur à un Comité de Protection des Personnes. L'avis de ce comité est notifié à l’autorité compétente par le promoteur avant le démarrage de la recherche.

11.3. Modifications

Le promoteur doit être informé de tout projet de modification du protocole par l’investigateur coordonnateur.

Les modifications devront être qualifiées en substantielles ou non.

Une modification substantielle est une modification susceptible, d'une manière ou d'une autre, de modifier les garanties apportées aux personnes qui se prêtent à la recherche biomédicale (modification d’un critère d’inclusion, prolongation d’une durée d’inclusion, participation de nouveaux centres,…).

Après le commencement de la recherche, toute modification substantielle de celle-ci à l’initiative du promoteur doit obtenir, préalablement à sa mise en oeuvre, un avis favorable du comité et une autorisation de l’autorité compétente. Dans ce cas, si cela est nécessaire, le comité s’assure qu’un nouveau consentement des personnes participant à la recherche est bien recueilli.

Par ailleurs, toute extension de la recherche (modification profonde du schéma thérapeutique ou des populations incluses, prolongation des traitements et ou des actes thérapeutiques non prévus initialement dans le protocole) devra être considérée comme une nouvelle recherche.

Toute modification substantielle devra faire l’objet **par le promoteur** **après paiement d’une taxe** d’une demande d’autorisation auprès de la DGS et/ou d’une demande d’avis du CPP.

11.4. Déclaration CNIL

La loi prévoit que la déclaration du fichier informatisé des données personnelles collectées pour la recherche doit être faite avant le début effectif de la recherche.

Une **méthodologie de référence spécifique au traitement de données personnelles opéré dans le cadre des recherches biomédicales définies par la loi 2004-806 du 9 août 2004** car entrant dans le champ des articles L.1XIV1-1 et suivants du Code de Santé Publique a été établie par la CNIL en janvier 2006.

Cette méthodologie permet une **procédure de déclaration simplifiée** lorsque la nature des données recueillies dans la recherche est compatible avec la liste prévue par la CNIL dans son document de référence.

Lorsque le protocole bénéficie d’un contrôle qualité des données par un ARC représentant le promoteur et qu’il entre dans le champ d’application de la procédure simplifiée CNIL, le promoteur demandera au responsable du fichier informatique de s’engager par écrit sur le respect de la méthodologie de référence MR06001 simplifiée.

**11.5. Note d’information et Consentement éclairé**

Modalités du recueil du consentement : le consentement sera recueilli lors de la visite pré-opératoire.

## Le consentement est présenté dans l’annexe 1.

### 11.6. Rapport final de la recherche

Le rapport final de la recherche sera écrit en collaboration par le coordonnateur et le biostatisticien pour cette recherche. Ce rapport sera soumis à chacun des investigateurs pour avis. Une fois qu'un consensus aura été obtenu, la version finale devra être avalisée par la signature de chacun des investigateurs et adressée au promoteur dans les meilleurs délais après la fin effective de la recherche. Un rapport rédigé selon le plan de référence de l’autorité compétente doit être transmis à l’autorité compétente ainsi qu’au CPP dans un délai de un an, après la fin de la recherche, s’entendant comme la dernière visite de suivi du dernier sujet inclus. Ce délai est rapporté à 90 jours en cas d’arrêt prématuré de la recherche.

12. TRAITEMENT DES DONNEES ET CONSERVATION DES DOCUMENTS ET DES DONNEES RELATIVES A LA RECHERCHE

Les documents d’une recherche entrant dans le cadre de la loi sur les recherches biomédicales doivent être archivés par toutes les parties pendant une durée de 15 ans après la fin de la recherche*.*

cet archivage indexé comporte :

- Les copies de courrier d’autorisation de l’Afssaps et de l’avis obligatoire du CPP
- Les versions successives du protocole (identifiées par le n° de version et la date de version),
- Les courriers de correspondance avec le promoteur,
- Les consentements signés des sujets sous pli cacheté (dans le cas de sujets mineurs signés par les titulaires de l’autorité parentale) avec la liste ou registre d’inclusion en correspondance,
- Le cahier d’observation complété et validé de chaque sujet inclus,
- Toutes les annexes spécifiques à l’étude,
- Le rapport final de l’étude provenant de l’analyse statistique et du contrôle qualité de l’étude (double transmis au promoteur).
- Les certificats d’audit éventuels réalisés au cours de la recherche

La base de données ayant donné lieu à l’analyse statistique doit aussi faire l’objet d’archivage par le responsable de l’analyse (support papier ou informatique).

**13. CONSIDERATIONS PHARMACO-ECONOMIQUES**

Il sera effectué une analyse des coûts du traitement et des réductions éventuelles de coût concernant les actes de radiologie interventionnelle, les examens d’imagerie, les traitements médicamenteux reçus (antibiotiques, antalgiques) ainsi que la durée d’hospitalisation.

14. ASSURANCE ET ENGAGEMENT SCIENTIFIQUE

### 13.1. Assurance

L'Assistance Publique- Hôpitaux de Paris est le promoteur de cette recherche. En accord avec la loi sur les recherches biomédicales, elle a pris une assurance auprès de la compagnie Biomedic-insurrance pour toute la durée de la recherche, garantissant sa propre responsabilité civile ainsi que celle de tout intervenant (médecin ou personnel impliqué dans la réalisation de la recherche) (loi n°2004-806, Art L.1121-10 du CSP).

Le promoteur se réserve le droit d'interrompre la recherche à tout moment pour des raisons médicales ou administratives; dans cette éventualité, une notification sera fournie à l'investigateur.

### 13.2. Engagement scientifique

Chaque investigateur s'engagera à respecter les obligations de la loi et à mener la recherche selon les B.P.C., en respectant les termes de la déclaration d'Helsinki en vigueur. Pour ce faire, un exemplaire de **l’engagement scientifique** daté et signé **par chaque investigateur** de chaque service clinique d’un centre participant sera remis au représentant du promoteur.

13.3. Règles relatives à la publication

Le comité des investigateurs est propriétaire des données et aucune utilisation ou transmission à un tiers ne peut être effectué sans son accord préalable.

seront premiers signataires des publications, les personnes ayant réellement participé à l’élaboration du protocole et son déroulement ainsi qu’à la rédaction des résultats.

Un comité d’écriture sera constitué et l’ordre des signataires pourra être défini par avance.

L’ Alliance pour la recherche en Cancérologie (APREC), doit être mentionnée comme étant le promoteur de la recherche biomédicale et comme soutien financier le cas échéant.

**15. BIBLIOGRAPHIE**

1. Harris AG. Future medical prospects for Sandostatin. Metabolism 1990;39(2):180–185

2. Ulibarri JI, Sanz Y, Fuentes C, et al. Reduction of lymphorrhagia from ruptured thoracic duct by somatostatin. Lancet 1990;336:258.

3. Schmid et al AACE 2005 présentation

3. Carcoforo P, Soliani G, Maestroni U, Donini A, Inderbitzin D, Hui TT, Lefor A, Avital I, Navarra G. Octreotide in the treatment of lymphorrhea after axillary node dissection: a prospective randomized controlled trial. J Am Coll Surg. 2003 Mar;196(3):365-9.

4. Mahmoud SA, Abdel-Elah K, Eldesoky AH, El-Awady SI. Octreotide can control lymphorrhea after axillary node dissection in mastectomy operations. Breast J. 2007 Jan-Feb;13(1):108-9.

5. Capocasale E, Busi N, Valle RD, Mazzoni MP, Bignardi L, Maggiore U, Buzio C, Sianesi M. Octreotide in the treatment of lymphorrhea after renal transplantation: a preliminary experience. Transplant Proc. 2006 May;38(4):1047-8.

7. Schmid HA, Schoeffter P. Functional activity of the multiligand analog SOM230 at human recombinant somatostatin receptor subtypes supports its usefulness in neuroendocrine tumors. Neuroendocrinology. 2004;80 Suppl 1:47-50.

8. Bruns C, Lewis I, Briner U, Meno-Tetang G, Weckbecker G. SOM230: a novel somatostatin peptidomimetic with broad somatotropin release inhibiting factor (SRIF) receptor binding and a unique antisecretory profile. Eur J Endocrinol. 2002 May;146(5):707-16.

9. Matulonis UA, Seiden MV, Roche M, Krasner C, Fuller AF, Atkinson T, Kornblith A, Penson R. Long-acting octreotide for the treatment and symptomatic relief of bowel obstruction in advanced ovarian cancer. J Pain Symptom Manage. 2005 Dec;30(6):563-9.

10. Mangili G, Franchi M, Mariani A, Zanaboni F, Rabaiotti E, Frigerio L, Bolis PF, Ferrari A. Octreotide in the management of bowel obstruction in terminal ovarian cancer. Gynecol Oncol. 1996 Jun;61(3):345-8.

11. Massacesi C, Galeazzi G. Sustained release octreotide may have a role in the treatment of malignant bowel obstruction. Palliat Med. 2006 Oct;20(7):715-6.

12. Mystakidou K, Tsilika E, Kalaidopoulou O, Chondros K, Georgaki S, Papadimitriou L. Comparison of octreotide administration vs conservative treatment in the management of inoperable bowel obstruction in patients with far advanced cancer: a randomized, double- blind, controlled clinical trial. Anticancer Res. 2002 Mar-Apr;22(2B):1187-92.

INFORMATION ET CONSENTEMENT ÉCLAIRÉ DE PARTICIPATION

À L'ÉTUDE

**« ÉVALUATION DE L’INTERET DE L’ADMINISTRATION DE PASIREOTIDE (SOM 230®) DANS LA PREVENTION DES LYMPHOCELES APRES CURAGE AXILLAIRE»**

Madame,

Vous présentez un cancer du sein pour lequel vous devez avoir une intervention chirurgicale. Au cours de cette intervention, le chirurgien procédera à l’ablation des ganglions axillaires. Ces ganglions sont reliés entre eux par des canaux lymphatiques. En période postopératoire, de la lymphe peut continuer à s’écouler des canaux sectionnés lors du curage. C’est pour cette raison que votre chirurgien met en place à la fin de l’intervention un drain au contact de la zone de curage. Malgré cette précaution, il peut survenir une collection au contact des vaisseaux appelée lymphocèle qui peut nécessiter une évacuation (par ponction) si celle-ci est responsable de symptômes cliniques.

L’octréotide est une hormone utilisée en chirurgie digestive dans la prévention postopératoire des fistules pancréatiques (écoulement de liquide pancréatique à partir du site opératoire) et dans la régulation des fistules digestives (permettant une diminution des sécrétions du tube digestif). La pasireotide (SOM 230®) est un analogue de la sansostatine possédant une affinité supérieure à l’octréotide sur quatre des cinq récepteurs à la somatostatine.

Plusieurs études récentes ont mis en évidence une diminution de l’incidence et de la nécessité des ponctions de lymphocèles postopératoire après réalisation d’un curage axillaire dans le cadre de la chirurgie d’un cancer du sein grâce à l’administration d’octréotide.

# Objectifs de l'étude

L'étude à laquelle nous vous proposons de participer à pour but de vérifier l'efficacité de l’administration systématique en post-opératoire d’un médicament, la SOM 230®, sur la réduction de l’incidence des lymphocèles symptomatiques.

# Déroulement de l'étude

Si vous acceptez de participer à cette étude, vous recevrez une injection intra-musculaire de SOM 230® LP 5 à 10 jours avant l’intervention.

# Bénéfices et complications attendus

Le risque lié à l’administration de ce médicament est en théorie nul puisque aucun effet secondaire n’a été rapporté dans son utilisation répétée dans d’autres indications. Seule des intolérances au glucose ont été rapportées et une mesure de la glycémie est effectué pour vérifier la tolérance après la première injection. Le bénéfice attendu est principalement une réduction des lymphocèles symptomatiques (qui nécessitent une ponction) et secondairement une diminution de la durée de drainage et du débit journalier des drains.

# Assurances

Pour la réalisation de cette étude et assurer les patientes en cas de complications une assurance a été souscrite.

# Confidentialité, surveillance et droits du patient

Les données recueillies lors de cette étude sont soumises au secret médical et demeureront strictement confidentielles.

La surveillance après la chirurgie sera assurée par votre chirurgien au cours des consultations prévues. Bien entendu, votre chirurgien sera à votre disposition, si toute complication ou événement intercurrent survenait entre deux consultations.

Vous êtes libre d'accepter ou de refuser de participer à cette étude et de retirer, à tout moment, votre consentement à participer à cette recherche et cela quelles que soient vos raisons et sans supporter aucune responsabilité.

**Le docteur m'a proposé de participer à la recherche biomédicale intitulée « ÉVALUATION DE L’INTERET DE L’ADMINISTRATION DE PASIREOTIDE (SOM 230®) DANS LA PREVENTION DES LYMPHOCELES APRES CURAGE AXILLAIRE »**

Le médecin m'a précisé que j'étais libre d'accepter ou de refuser de participer à cette étude.

L'étude à laquelle on me propose de participer à pour but d'évaluer un moyen de prévention des lymphocèles symptomatiques postopératoire. Mon chirurgien souhaite évaluer l’efficacité de la SOM 230® dans la diminution des lymphocèles postopératoire.

Afin d'éclairer ma décision, j'ai bien compris que cette étude cherche à évaluer l’efficacité de la SOM 230® dans cette indication. J'accepte que les données enregistrées à l'occasion de cette étude puissent faire l'objet d'un traitement informatisé ou d'une publication scientifique. Les données recueillies sont soumises au secret médical et demeureront strictement confidentielles.

Je suis parfaitement consciente que je peux retirer à tout moment mon consentement à ma participation à cette recherche et cela quelles que soient mes raisons et sans supporter aucune responsabilité.

Après en avoir discuté et avoir obtenu réponse à toutes mes questions, j'accepte librement et volontairement de participer à la recherche décrite ci-dessus.

Fait à , le

Personne donnant le consentement: Médecin investigateur:

Nom, prénom: Nom, prénom:

Signature: Signature:

**Budget :**

Assurance : 7500 euros

1/2 temps TEC TENON 22500 euros

1/2 temps TEC IGR 22500 euros

1/2 temps monitorage : ARC 22500 euros

Biostatistique 15000 euros

Réalisation / impression des CRF (dupli) :

90 CRF = 5000 euros

CPP / AFSSAPS : 1000 euros

Soins infirmiers injections :

acte = 18,15 euros (1 actes infirmier par patient) 1750 euros

Déplacement ARC/TEC/investigateurs 3000 euros

Frais logistique promoteur 7500 euros

**Total 108250 euros**
