# Supplementary material for: Evaluation of the Effects of Pasireotide LAR Administration on Lymphocele Prevention after Axillary Node Dissection for Breast Cancer: Results of a Randomized Non-Comparative Phase 2 Study
Source: PLoS One. 2016 Jun 9;11(6):e0156096. doi: 10.1371/journal.pone.0156096 (PMC4900597; doi:10.1371/journal.pone.0156096)
Supplement: S4 File — (DOCX) [file pone.0156096.s004.docx]

**Statistical analysis of the primary outcome**

Statistical analysis of included patients was performed sequentially, after each observed response (success, failure) of included patient in each arm (pasireotide or placebo). It consists in sequentially estimating the probability of success (i.e., patients who did not develop post-operative axillary symptomatic lymphoceles was considered as success) by using a Bayesian approach, based on a beta-binomial model (Zohar 2008).

This approach is based on updating after each inclusion, in each group, of our base line knowledge on the success rate, which is prior to the onset of trial. It thus requires specifying a probability model for the success rate, denoted$\pi_{pa}$ (in the pasireotide arm) and $\pi_{pl}$ (in the placebo arm), before the trial onset. We choose a beta (*a*, *b*) model, with mean fixed at 80% (i.e., the supposed success probability) in the pasireotide arm and 60% in the placebo arm according to the investigators’ initial opinion. Using Bayesian inference allows us to include subjective opinion in the prior distribution and to analyse it according to each prior belief. In this trial we have choose two types of informative priors in each arm, prior 1 is less informative than prior 2. The distributions of probabilities of success in each arm are given in figure 1.


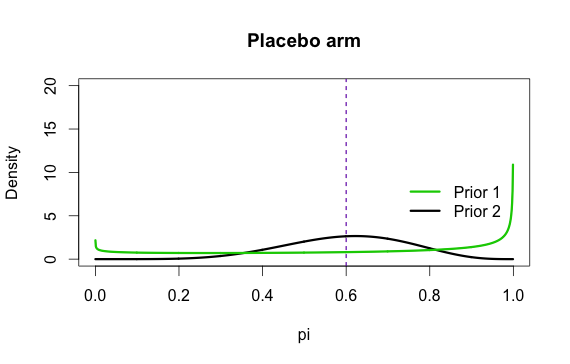

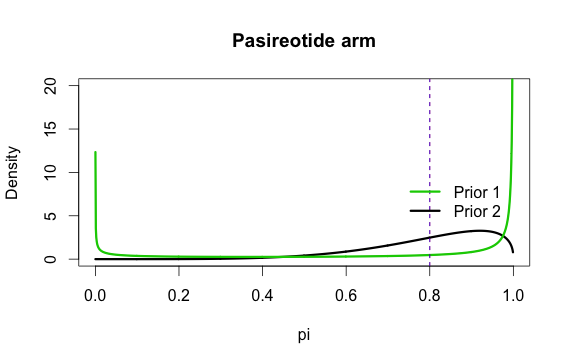


Figure 1 – Prior distributions in each arm. Prior 1 is less informative than prior 2 in each arm. In the pasireotide arm both priors are centred on 80% but the variance differs between prior distributions (prior 1: Beta(0.48,0.12) and prior 2: Beta(5.6,1.4)) and in the placebo arm both priors centred on 60% but the variance differs between prior distributions (prior 1: Beta(0.84,0.56) and prior 2: Beta(6.4,4.4))

The prior densities in each arm were updated after each inclusion, generating a so-called posterior beta-binomial distribution; the mean of which is given by ${E(\pi}_{i})=\frac{a_{i}+r_{i}}{b_{i}+n_{i}-r_{i}}$where *i=pa* or *pl*, *r_i_* is the number of observed successes and *n_i_* the number of patients recruited so far.


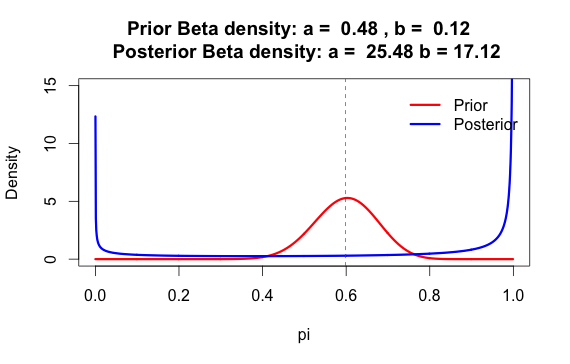

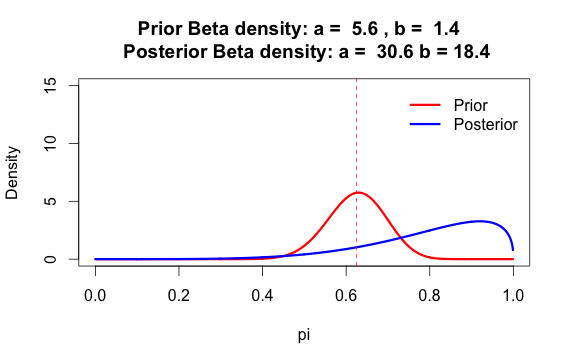


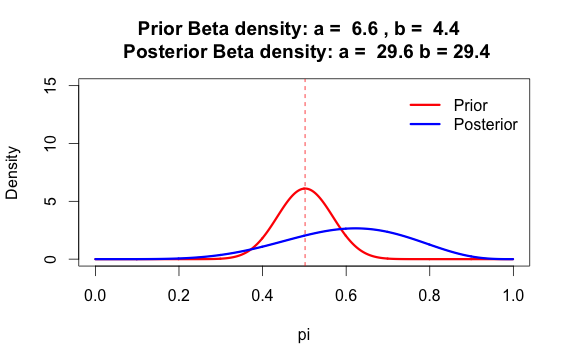

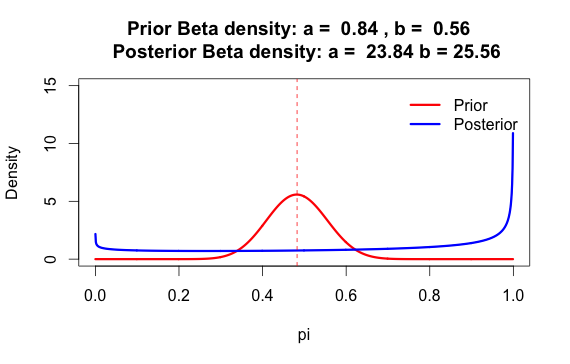
Figure 2 – Posterior distribution for the pasireotide arm. On the left posterior according to prior 1 and on the right posterior according to prior 2.

Figure 3 – Posterior distribution for the placebo arm. On the left posterior according to prior 1 and on the right posterior according to prior 2

Forty-two patients were included in the pasireotide arm where 25 successes were observed and 48 patients were included in the placebo arm where 23 responses were observed (Figure 2 and 3). In table 1 are given the estimation of the mean posterior of success rate in each arm according to each type of prior. In the paper are given the results for each arm according to the type of prior 2.

Table 1: Estimated mean posterior of success rate for each arm according to each prior type.

|  | **Pasireotide** | | **Placebo** | |
| --- | --- | --- | --- | --- |
|  | Prior 1 | **Prior 2** | Prior 1 | **Prior 2** |
|  | Beta (0.48,0.12) | **Beta (5.6,1.4)** | Beta (0.84,0.56) | **Beta (6.6,4.4)** |
| **N** | 42 | **42** | 48 | **48** |
| **Nb of successess** | 25 | **25** | 23 | **23** |
| **Estimated mean posterior of success rate** | 59.8% | **62.4%** | 48.3% | **50.2%** |
| **95% Credibility Interval** | 44.9%-73.8% | **48.6%-75.3%** | 34.6%-62.1% | **37.6%-62.8%** |

Ref : Bayesian design and conduct of phase II single-arm clinical trials with binary outcomes: a tutorial. Zohar S, Teramukai S, Zhou Y. Contemp Clin Trials. 2008 Jul;29(4):608-16
